# Anti-PD-1 Immunotherapy in Preclinical GL261 Glioblastoma: Influence of Therapeutic Parameters and Non-Invasive Response Biomarker Assessment with MRSI-Based Approaches

**DOI:** 10.3390/ijms21228775

**Published:** 2020-11-20

**Authors:** Shuang Wu, Pilar Calero-Pérez, Carles Arús, Ana Paula Candiota

**Affiliations:** 1 Departament de Bioquímica i Biologia Molecular, Unitat de Bioquímica de Biociències, Edifici Cs, Universitat Autònoma de Barcelona, 08193 Cerdanyola del Vallès, Spain; wu.shuang@uab.cat (S.W.); Pilar.Calero@uab.cat (P.C.-P.); carles.arus@uab.cat (C.A.); 2 Centro de Investigación Biomédica en Red en Bioingeniería, Biomateriales y Nanomedicina (CIBER-BBN), 09183 Cerdanyola del Vallès, Spain; 3 Institut de Biotecnologia i de Biomedicina (IBB), Universitat Autònoma de Barcelona, 08193 Cerdanyola del Vallès, Spain

**Keywords:** glioblastoma, orthotopic immunocompetent tumours, checkpoint inhibitors, TMZ, therapy response biomarker, magnetic resonance spectroscopic imaging, long-term immune memory

## Abstract

Glioblastomas (GBs) are malignant brain tumours with poor prognosis even after aggressive therapy. Programmed cell death-1 (PD-1) immune checkpoint blockade is a promising strategy in many types of cancer, but its therapeutic effects in GB remain low and associated with immune infiltration. Previous work suggests that oscillations of magnetic resonance spectroscopic imaging (MRSI)-based response pattern with chemotherapy could act as a biomarker of efficient immune system attack onto GBs. The presence of such oscillations with other monotherapies such as anti-PD-1 would reinforce its monitoring potential. Here, we confirm that the oscillatory behaviour of the response biomarker is also detected in mice treated with anti PD-1 immunotherapy both in combination with temozolomide and as monotherapy. This indicates that the spectral pattern changes observed during therapy response are shared by different therapeutic strategies, provided the host immune system is elicited and able to productively attack tumour cells. Moreover, the participation of the immune system in response is also supported by the rate of cured animals observed with different therapeutic strategies (in the range of 50–100% depending on the treatment), which also held long-term immune memory against tumour cells re-challenge. Taken together, our findings open the way for a translational use of the MRSI-based biomarker in patient-tailored GB therapy, including immunotherapy, for which reliable non-invasive biomarkers are still missing.

## 1. Introduction

Glioblastoma (GB) is the most common primary malignant tumour of the central nervous system (CNS) in adults. Clinically, the 5-year survival rate of patients with GB is less than 9%, and the median survival time of them is only around 15 months despite the availability of multimodal therapies [1]. Standard therapy for GB is composed of surgical resection, radiotherapy and adjuvant chemotherapy with Temozolomide (TMZ), but this standard schedule does not prevent tumour recurrence, mostly due to the infiltrative growth pattern, and chemoresistance [2,3].

In recent years, an increasing number of studies indicated that immune escape has played an important role in tumour progression [4,5,6]. In other words, resistance to chemotherapy is not only related to the intrinsic properties of tumour cells but also to other parameters of the tumour microenvironment [7]. Tumours have evolved in multiple ways to evade the immune system by creating a strong immunosuppressive microenvironment, which protects tumour cells from the immune attack by exploiting inhibitory immune checkpoints such as the programmed cell death ligand-1 (PD-L1)/programmed cell death-1 (PD-1) axis, using check point inhibitors (CKI) [8]. Ultimately, the final phase of escape from immune surveillance allows tumour cells to thrive unchecked in an immunosuppressive tumour microenvironment.

With the rapid development of immunotherapy, several anti PD-1 antibodies are being extensively used in clinics. These were approved in the treatment of melanoma, non-small cell lung cancer, renal cell cancer, Hodgkin’s lymphoma, and bladder cancer [9,10,11,12]. Moreover, anti-PD-1 immunotherapeutic approaches are currently undergoing clinical study for safety and efficacy in GB [13], with some success when used in a neoadjuvant approach [13,14]. Unfortunately, the combination therapy of anti-PD-1 and classical radiotherapy does not seem to provide benefit for progression free survival (PFS) in the interim report of the CheckMate-548 Phase 3 clinical trial [15]. This indicates that additional preclinical work may be needed to optimize CKI potential for GB treatment in patients. In this respect, preclinical studies revealed that anti-PD-1 monotherapy exhibits considerable potential for enhancing an anti-tumour immune response in the GL261 immune-competent mouse model [16,17,18]. However, the overall survival (OSs) of mice under anti-PD-1 monotherapy was quite variable among those studies, possibly due to their different dosing scheme and therapy starting days (i.e., small, large tumours). In this sense, we found it relevant to assess the impact of initial tumour volume and dosing schedule on the anti-PD-1 monotherapy efficacy. In addition, immune checkpoint inhibitors combined with the gold standard chemotherapy (TMZ) could represent another way to enhance treatment efficiency for GB. In preclinical studies, the combination of anti PD-1 antibody and localized radiation therapy has proved to prolong survival in a GL261 immune-competent mouse model [17]. Moreover, the combination therapy of anti-PD-1 antibody and chemotherapeutic drug (carmustine) greatly shrank tumour size and improved survival rate in the GL261 immune-competent mouse model [19]. Our previous work [20] found that PD-L1 content was significantly increased in late relapsing TMZ-treated tumours compared to vehicle-treated tumours, which reveals that the upregulated expression of PD-L1 on tumour cells could be a potential link between chemotherapy and tumour immune resistance. Therefore, we should expect an improved therapeutic potency when anti-PD-1 treatment is combined with TMZ in an immune-enhancing metronomic schedule (IMS) in GL261 immune-competent mice.

Magnetic resonance imaging (MRI) plays a crucial role in GB detection, treatment planning, and therapy response assessment, evaluating tumour presence, size and characteristics in a non-invasive way [21]. MRI provides anatomical information and other data such as contrast enhancement or biophysical parameters (e.g., perfusion). On the other hand, magnetic resonance spectroscopy, or its multivolume variation, spectroscopic imaging (MRSI), provides additional information about millimolar concentrations of low molecular weight metabolites in the studied tissue [22,23,24], which can inform about its biochemical/molecular environment. Previous studies from our group suggest that the proper analysis of such metabolomic information could give hints about tumour response before changes are observed in tumour size [25,26]. Given TMZ administered in an IMS has produced an oscillatory pattern of response sampled by MRSI-based nosological images, and this oscillatory pattern of response has been proposed to behave as an immune system efficiency biomarker during GB therapy follow-up [20]. In this study, we checked the robustness of the biomarker under different therapeutic options, namely the presence of an oscillatory pattern of response to therapy in tumours treated with IMS-anti-PD-1 monotherapy and IMS-TMZ/anti-PD-1 combination therapy.

In the present clinical landscape, patients with GB always relapse even after the best accepted therapeutic protocols are applied, including surgical resection. Accordingly, both response improvement and the generation of tumour-specific immunological memory would be crucial to improve prognosis. Thus, we tested how the different treatment modalities had an impact on tumour recurrence after curing mice and whether MRSI-based nosological images previously developed to monitor the response to TMZ treatment could also reflect tumour response to immunotherapeutic strategies, acting as a generic surrogate biomarker of therapy response.

## 2. Results

Results reflect first the methodologic steps taken in order to achieve the best protocol for immunotherapy. This means first to produce proper response to therapy, and from tumours large enough to acquire data for non-invasive MRSI-based biomarker therapy response determination. Secondly, results also comprise the evaluation of the MRSI-based nosological images along treatment time, in order to check whether we were able to non-invasively assess response with immunotherapeutic approaches, either alone or in combination with TMZ.

### 2.1. IMS-TMZ/anti-PD-1 Combined Therapy Is Superior to Monotherapy in Orthotopic GL261 Glioma Bearing Mice

**Figure 1:**
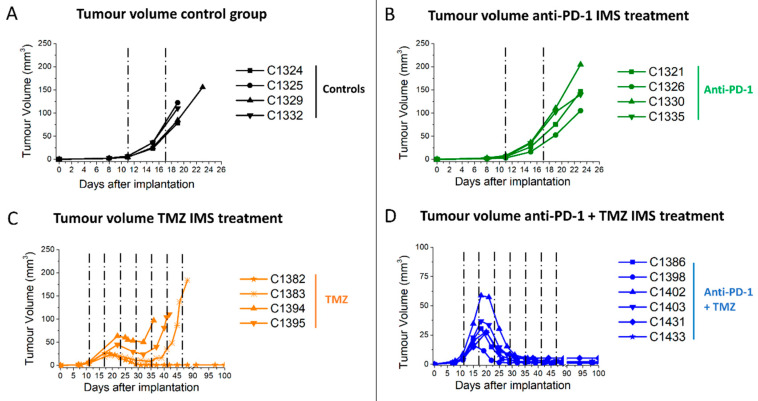
Tumour volume evolution of mice in monotherapy and combination therapy groups. (**A**) Tumour volume evolution of mice treated with (**A**) control isotype murine immunoglobulin G (IgG) in immune-enhancing metronomic schedule (IMS) (*n* = 4, black lines); (**B**) anti-PD-1 in IMS (*n* = 4, green lines); (**C**) Temozolomide (TMZ) alone in IMS (*n* = 4, orange lines); and (**D**) anti-PD-1 combined with TMZ in IMS (*n* = 6, blue lines). The black dashed lines indicate therapy administration time points. Cxxxx corresponds to a unique alpha-numeric animal identifier code in the *Grup d’Aplicacions Biomèdiques de la Ressonància Magnètica Nuclear* (GABRMN) group.

Four treatments in IMS (every 6 days) were conducted as follows: isotype immunoglobulin G (IgG) control (100 μg/day, *n* = 4), anti-PD-1 (100 μg/day, *n* = 4), TMZ (60 mg/kg, *n* = 4) and a combination of anti-PD-1 and TMZ (100 μg/day and 60 mg/kg, *n* = 6). Mice tumour volume and body weight were inspected twice a week (Figure 1 and Figure S1).

**Figure 2:**
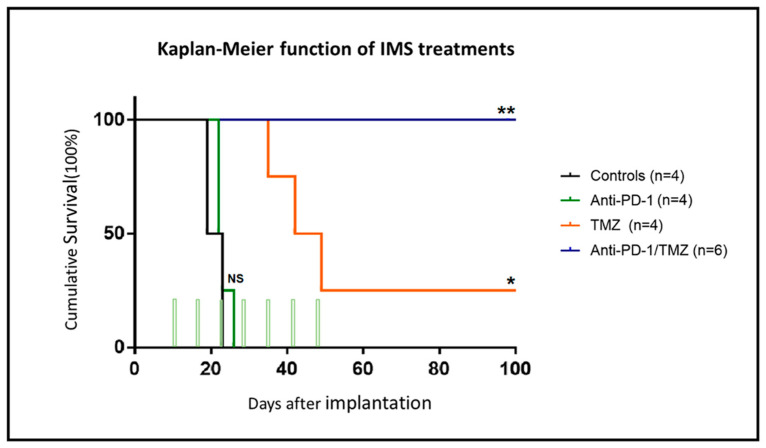
Survival Kaplan–Meier curves of mice in different therapy groups. Mice treated with control isotype murine IgG 100 µg/day (*n* = 4, black line), anti-PD-1 100 µg/day (*n* = 4, green line), anti-PD-1 100 µg/day combined with TMZ 60 mg/kg (*n* = 5, blue line) and TMZ 60 mg/kg alone (*n* = 4, orange lines), the green columns indicating therapy administration time points. Note: mouse C1403 (combination therapy group) died on day 98 p.i. during the re-challenge experiment (not recovering from anaesthesia). MRI scanning did not show a relapsing tumour; accordingly, GB did not cause its death, and this individual was excluded from the survival curve calculations since it died from unknown reasons. ** *p* < 0.01, * *p* < 0.05 and NS = no significant difference for the Log-Rank test for the comparison between treated groups and control group.

Tumours on anti-PD-1 monotherapy (100 μg/day) group and on isotype murine IgG control (100 μg/day) presented similar results with no apparent response to therapy. Tumours in the TMZ monotherapy group showed transient shrinkage followed by relapse, except in one case (C1382) in the TMZ group which was cured after six doses of TMZ treatment. Notably, all mice in the anti-PD-1/TMZ combined therapy group were declared cured, i.e., all tumours were reduced to a stable tissue scar for one month and the mice were re-challenged (see Section 2.4). Tumour volume evolution was significantly different (*p* < 0.001) when comparing the control group with the TMZ monotherapy group and combined anti-PD-1/TMZ group. Moreover, Kaplan–Meier curves were elaborated (Figure 2) to compare the animal survival rate among the four groups.

For the anti-PD-1 treatment (100 µg/day), a survival rate of 23 ± 2.9 days was found, which was not significantly different neither from the isotype IgG control group (21.8 ± 3.3 days) nor from the untreated (control) mice previously studied in our group, 22.5 ± 3.0 days (*n* = 6) [20]. During the long-term survival observation period (100 days p.i.), the IMS–TMZ treatment led to a survival rate of 56.5 ± 29.6 days, which was significantly different and improved when compared with the anti-PD-1 monotherapy or control mice (*p* < 0.001). The best results were obtained with the combined anti-PD-1 and TMZ treatment, with a full survival rate of 100 ± 0 days (*n* = 5), which is significantly different (*p* = 0.02) from IMS–TMZ alone. Importantly, at the time of comparing the different treated groups, significant differences were found regarding mice body weight changes, tumour volume evolution and survival average, the best overall outcome always being obtained with the anti-PD-1/TMZ combined treatment in IMS, which proved clearly better than either monotherapy alone.

### 2.2. Initial Tumour Volume and Administration Schedule Are Critical for the Efficacy of Anti-PD-1 Monotherapy

Since there was no consensus in the literature (or even lack of information) regarding the initial tumour volume or dosing schedule when administering anti-PD-1 treatment to mice, we designed two series of experiments to assess these variables. The first series of experiments was designed to explore the impact of initial tumour size on treatment effect. Mice were divided into two groups according to the tumour size registered at day 11 post-implantation. As shown in Figure S2A, significant differences (*p* < 0.005) were found between the cases assigned to the large initial tumour group (*n* = 4, 9.6 ± 2.2 mm^3^) and the small initial tumour group (*n* = 4, 2.1 ± 1.1 mm^3^). The “normal initial tumour volume” was defined based on average GL261 GB tumour volumes studied by our group in preceding years, for example 5.4 ± 2.6 mm^3^ (*n* = 19) described in [20]. Therefore, the “large initial tumour volume” group included tumours with higher average volumes while the “small initial tumour volume” group included tumours with lower average volumes. Since the first low anti-PD-1 dosage attempted did not prove effective to increase mice survival (see Section 2.1, Figure 2), a new therapeutic schedule and dose was adapted from [16]. Thus, anti-PD-1 was administered from day 11 p.i. (500 μg/dose) followed by repeated injections (250 μg/dose) every 3 days. The experimental schedule and tumour T2w MRI image of representative cases on the therapy starting day are shown in Figure S2B.

The second series of experiments aimed to investigate the effect of changes in the dosing schedule while using similar tumour volumes. Mice bearing similar sized tumours were divided randomly into two groups on day 6 post-implantation, being 0.39 ± 0.19 mm^3^ in the group with a dosing schedule of every 3 days (E3D) and 0.37 ± 0.06 mm^3^ in the standard IMS (every 6 days) group. Figure S3A presents the tumour size distribution of each group, and no significant differences were found between groups (*p* > 0.05). In E3D group, anti-PD-1 was administered from day 6 p.i. (500 μg/dose) followed by repeated injections (250 μg/dose) every 3 days. In the IMS group, anti-PD-1 was administered from day 6 p.i. (500 μg/dose) followed by repeated injections (250 μg/dose) with IMS (every 6 days). The experimental schedule for each group is shown in Figure S3B.

#### 2.2.1. Initial Tumour Volume Effect

**Figure 3:**
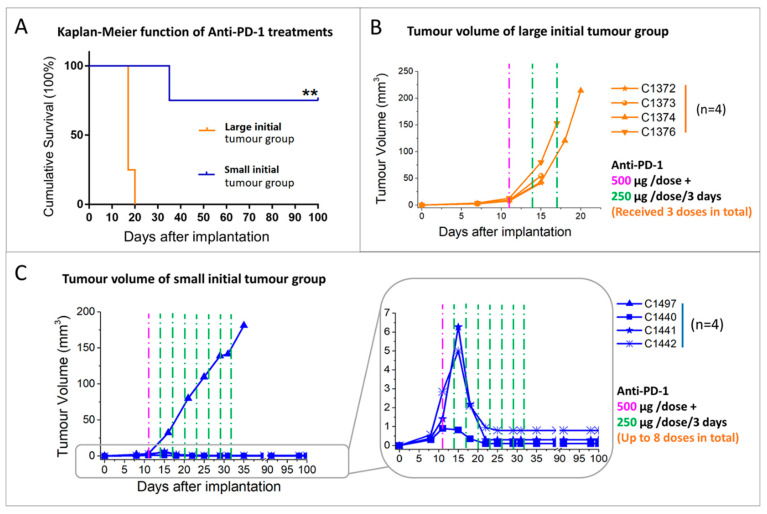
Mice bearing different sized tumours have been treated with the same anti-PD-1 dosing schedule. (**A**) survival Kaplan–Meier curves for mice bearing large tumours (*n* = 4, orange line) and small tumours (*n* = 4, blue line) treated with anti-PD-1 every 3 days; (**B**) tumour volume evolution of mice bearing large tumours treated with anti-PD-1 every 3 days (*n* = 4, orange lines); (**C**) tumour volume evolution of mice bearing small tumours treated with anti-PD-1 every 3 days (*n* = 4, blue lines). The purple and green dashed line (500 µg/dose and 250 µg/dose separately) indicate therapy administration time points. Inset at bottom right shows an expanded view of 3 of the 4 investigated tumours in C. ** *p* <0.01 for Log-Rank test for the comparison between different groups.

Under the higher dosage (500/250 μg) chosen for anti-PD-1 monotherapy at the E3D schedule, the mice survival rate was significantly different (*p* < 0.001) according to the tumour starting volumes. Survival was remarkably higher in the small initial tumour volume group (170 ± 90 days, 75% of mice cured) than in the large initial tumour group (17.8 ± 1.5 days, 0% cured) at the same dosing schedule, Figure 3A. The tumour growth curves (Figure 3B,C) demonstrated the regression of the tumour observed in most cases from the small initial tumour volume group.

#### 2.2.2. Administration Schedule Effect: Every 3 Days vs. 6 Days

**Figure 4:**
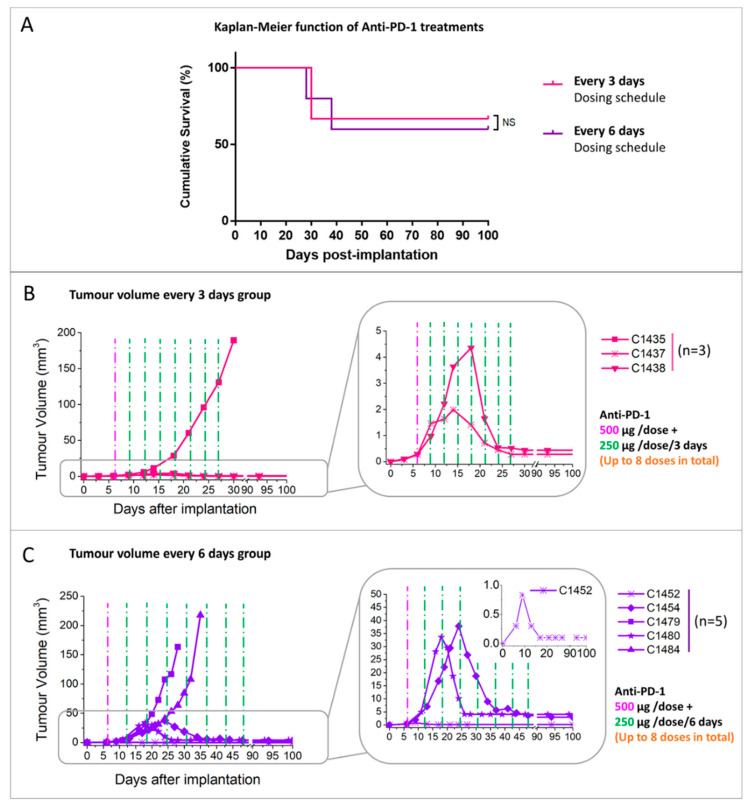
Mice bearing similar sized tumours on the therapy starting day and treated with different anti-PD-1 dosing schedules. (**A**) survival Kaplan–Meier curves for mice treated with anti-PD-1 every 3 days dosing schedule (*n* = 3, pink line) and IMS (*n* = 5, purple line); (**B**) tumour volume evolution of mice treated with anti-PD-1 every 3 days dosing schedule (*n* = 3, pink lines); (**C**) tumour volume evolution of mice treated with anti-PD-1 in IMS (*n* = 5, purple lines). The purple and green dashed line (500 µg/dose and 250 µg/dose separately) indicate therapy administration time points. Inset at bottom right shows expanded views of 3 of the 5 investigated tumours in C. NS = non-significant difference for the Log-Rank test for the comparison between different groups.

Regarding the series of mice bearing similar size tumour volumes on the therapy starting day treated with anti-PD-1 (500/250 μg) but using a different dosing schedule (i.e., E3D vs. IMS), no significant differences were found between the E3D group (91.3 ± 53.1 days) and IMS group (85.8 ± 49.6 days) (*p* > 0.05 Figure 4A). The tumour volume evolution plot for each group is shown under their respective anti-PD-1 dosing scheme (Figure 4B,C). The cure rate in the E3D group was 66.6% and that in the IMS group was 60.0%.

Results show that, regarding the two parameters investigated, the tumour volume at the therapy starting point is definitely a determinant for therapy outcome. On the other hand, and using the chosen dosage of 500/250 µg, no significant differences were observed when treating mice every 3 days or every 6 days, with a survival rate and percentage of cure being similar in both cases. In this sense, there would be no improvement with the increase in the anti-PD-1 frequency of administration and a 6-day schedule would be more suitable, since it produces the same results, consumes less therapeutic agent and produces less stress to animals. Accordingly, the IMS schedule and small initial volumes on the therapy starting day were chosen for further non-invasive biomarker assessment studies.

### 2.3. Multi-Slice MRSI-Based Volumetric Analysis under IMS-Anti-PD-1/TMZ Combined Treatment or IMS-Anti-PD1 Monotherapy: Non-Invasive Biomarker of Therapy Response

A longitudinal study was performed with *n* = 6 GL261 tumour bearing mice: three were treated with IMS-anti-PD-1/TMZ and three with IMS-anti-PD-1. Mice were studied every two days until endpoint, and the start of MRSI explorations was conditioned by the measured tumour volume. Results are summarized below.

#### 2.3.1. IMS-anti-PD-1/TMZ Treated Mouse

The results of the multi-slice MRSI-based volumetric analysis of GL261 GB treated with IMS-anti-PD-1/TMZ combination therapy of a representative, responding case C1446 is summarized below. Results for additional responding cases are described in the Supplementary Materials (Figures S4 and S5).

**Figure 5:**
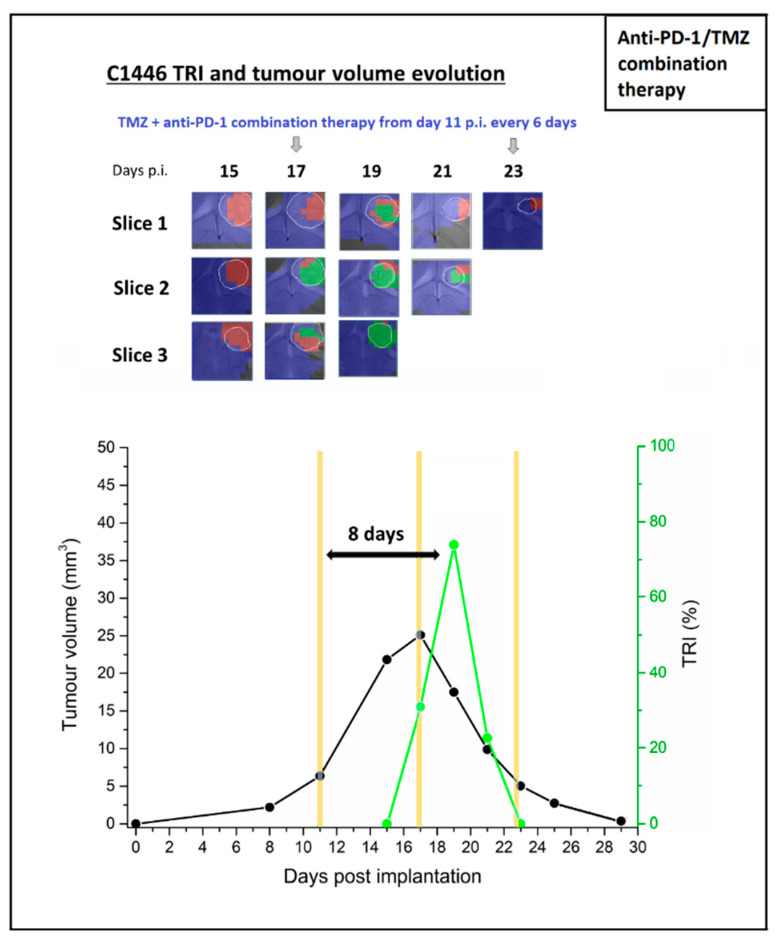
Nosological images and graphical representation of the tumour volume evolution for the tumour region in the case C1446. Tumour volume in mm^3^ (black line, left axis) and the percentage of green, responding pixels (tumour responding index (TRI)) obtained taking into account total pixels counting (green line, right axis). In the upper part of every image, chosen time points show the evolution of the nosological images in three rows of colour-coded grids superimposed to the T2w-MRI for each slice. Vertical arrows indicate days of therapy administration. In the bottom graph, yellow columns indicate anti-PD-1/TMZ combination therapy administration days. TRI cycle duration (therapy administration to next peak maxima) is highlighted in the image. The TRI peak appears 8 days after the first round of PD-1/TMZ administration.

C1446 was analysed by MRI and multi-slice 3D MRSI from day 15 until day 23 post-implantation. Therapy was administered at days 11, 17 and 23 p.i. right after MRSI analysis, and continued to be given in IMS until the animal was considered cured (day 41 p.i.). The relationship between tumour responding index (TRI) and tumour volume, as well as the corresponding nosological images, are shown in Figure 5.

In case C1446, TRI increased from 0% at day 15 p.i. to 74.2% at day 19 p.i. and at day 23 TRI decayed to 0% again. After this period, the tumour entered a “below threshold detection period” (BTDP) period, thus only one TRI peak could be observed. It is noteworthy that the tumour volume of C1446 reached the maximum size on day 17 p.i., when responding tumour pixels started to be observed. Along with the presence of the TRI peak, the tumour volume dropped by 78.9% from its maximum between day 17 p.i. and day 23 p.i., probably due to the combined action of TMZ and immune system attack on the tumour during this shrinkage. The outstanding effectiveness of the treatment prevented observing further TRI cycles, but the appearance of a TRI maximum at day 8 is within the range of values observed for IMS–TMZ oscillation periods.

#### 2.3.2. IMS-anti-PD-1 Monotherapy Treated Mice

The results of the multi-slice MRSI-based volumetric analysis of GL261 GB treated with anti-PD-1 monotherapy of a representative, responding case C1480 is summarized below. Additional cases with transient or no response are described in Supplementary Materials (Figures S6 and S7).

##### C1480 Anti-PD-1 Monotherapy

**Figure 6:**
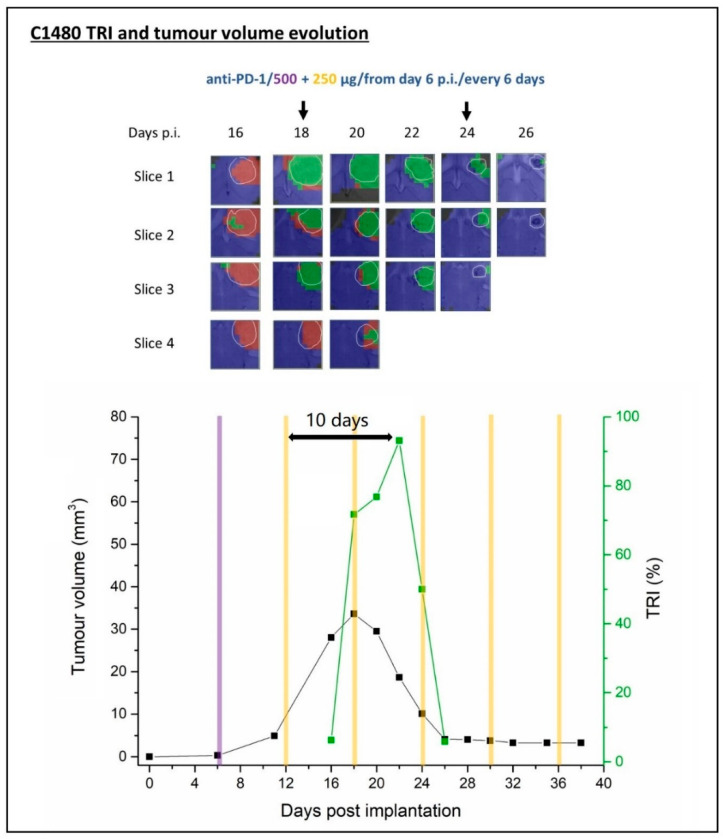
Nosological images and the graphical representation of the tumour volume evolution for the tumour region in the case C1480. Tumour volume in mm^3^ (black line, left axis) and the percentage of green, responding pixels (TRI) obtained taking into account the total pixels counting (green line, right axis). In the upper part of every image, the chosen time points show the evolution of the nosological images in two to four rows of colour-coded grids superimposed to the T2w-MRI for each slice. Vertical arrows indicate the days of therapy administration. In the bottom graph, the purple and yellow column (500 µg/dose and 250 µg/dose separately) indicate anti-PD-1 administration days. TRI cycle duration (therapy administration to next peak maxima) is highlighted in the image.

Case C1480 was analysed by MRI and multi-slice 3D MRSI from day 16 until day 26 p.i. MRSI acquisitions were forced to be stopped from day 26 p.i. since the C1480 tumour had shrunk down to a 4.12 mm^3^ scar, entering the BTDP. Therapy was administered on days 6, 12, 18, 24, 30 and 36 p.i., and was maintained in IMS until the animal was considered cured. The relationship between tumour volume and TRI evolution accompanied by the corresponding nosological images is shown in Figure 6.

In this case, the TRI increased from 6.3% at day 16 p.i. to 71.7% at day 18 p.i. and a further increase to 93.1% was measured at day 22 p.i. After this period, the tumour entered a BTDP period so only one clear TRI peak was observed. It is noteworthy that it is unclear whether the inflection point seen at day 20 p.i. has some biological meaning (i.e., a “pseudo” cycle), which would place the maximum TRI at 6 days from the administration point. However, the criteria set to decide whether a TRI change was a cycle or not does not agree with this possibility, and the maximum TRI was considered to be reached at day 22 p.i., 10 days after the second anti-PD-1 round.

The tumour volume of mouse C1480 grew until day 18 p.i., when abundant responding tumour pixels started to be observed. Then, along with the presence of a TRI peak, the tumour volume dropped 87.5% between day 18 p.i. and day 26 p.i., probably due to an immune system sustained attack onto the tumour leading to shrinkage. One clear TRI cycle was observed in this case, 10 days in length, which seemed slightly longer than the average observed with other therapy response TRI cycles. We cannot discard that a slower immune system build-up effect takes place from the effect at day 12 p.i. therapy administration time.

##### TRI Oscillations Were Generally Coincident with Response

Seven TRI oscillations were observed in the IMS-anti-PD-1/TMZ and IMS-anti-PD-1 treated mice and these were generally coincident with stable disease (SDi) and partial response (Pre) tumour stages (Table S1), according to the adapted Response Evaluation Criteria in Solid Tumours (RECIST) criteria. It is also worth noting that even when tumour volume evolution would point to PD, TRI oscillation may point to transient response (e.g., case 1484 day 18 p.i., see Figure S6), reinforcing the idea that the information provided by MRSI appears in an early/differential fashion compared with the response evaluated by tumour volume changes.

##### MRSI Spectral Quality

The MR spectra acquired were of overall good quality and examples of chosen MRSI spectra for cases C1480 (IMS-anti-PD-1 treated) and C1446 (IMS-anti-PD-1/TMZ treated) are shown in Figure S8. Major metabolites are identified in spectra classified as normal brain parenchyma, actively proliferating tumour or responding tumour. The good spectral quality, which was checked consistently in order to discard the presence of artifacts, ensures that differences detected with pattern recognition methods are not random or attributed to a data lack of quality. This allows us to be confident in the non-invasive assessment of therapy response through MRSI-based nosological imaging based in the metabolomics pattern.

### 2.4. Anti-PD-1 Monotherapy Shown to Be More Effective in Establishing Anti-Tumour Immune Memory than Combined Therapy

From the aforementioned work, fourteen mice had tumours which disappeared after TMZ and/or anti-PD-1 treatment (*n* = 6 in the IMS-TMZ/PD-1 combination group, and *n* = 8 in the anti-PD-1 monotherapy group, see Table 1 for individual codes).

Although curing GL261 GB-bearing animals was an outstanding achievement during this work, there was a great interest to assess whether treatments could lead to an immune memory that would prevent the further development of the same type of tumours, strengthening the idea that the non-invasive biomarker was somewhat linked to the immune system local action. To investigate whether IMS–TMZ therapy, IMS-anti-PD-1/TMZ combined therapy or anti-PD-1 monotherapy could produce such immune memory, we performed tumour re-challenge studies with the reimplantation of GL261 cells in the opposite side. Magnetic resonance imaging acquisitions (volumetric T2w) were acquired twice a week to check for tumour development and 3 wt C57BL/6 mice were implanted in parallel as controls. Figure S9 shows the tumour volume evolution after the re-challenge experiment.

#### 2.4.1. Control Mice

Mice in the wt control group had a 0% tumour rejection rate and died by day 21 post-implantation. No significant differences were found when compared to the control mice from our group, 19.7 ± 2.7 days. In addition, control tumours grew normally and no significant differences were found in comparison with a standard GL261 tumour doubling time of 2.4 ± 0.3 days [27].

#### 2.4.2. IMS-Anti-PD-1/TMZ Cured Mice

Regarding the cured mice from IMS-Anti-PD-1/TMZ combined therapy group, three (C1402, C1431 and C1433) out of six tumours (50%) re-implanted in the cured mice grew after 6 days. Anti-PD-1/TMZ combined therapy was administered as usual in IMS immediately after regrowth detection. One mouse (C1402) showed a transient response with tumour shrinkage but eventually died on day 38 post re-challenge, while the remaining (C1431 and C1433) were cured again after 2 doses of anti-PD-1/TMZ therapy. Therefore, 3/6 of the combined treatment showed “suboptimal memory”, and the initial tumour rejection rate of IMS-anti-PD-1/TMZ cured mice was 50% (3/6).

#### 2.4.3. Anti-PD-1 Monotherapy Cured Mice

Cured mice that had received anti-PD-1 monotherapy exhibited the best tumour rejection rate (8/8, 100%), and all of them displayed optimal immune memory, with no tumour growth detectable in any of the mice after the re-challenge experiment.

As summarised in Table 2, the re-challenge survival experiments demonstrated that different therapies exhibited varying degrees (50–100%) of long-term protective immune memory development against the further development of GL261 GB.

## 3. Discussion

### 3.1. Harnessing the Immune System to Control GB Tumour Progression

As a natural anti-tumour defence system, the immune system plays an important role in the response to anti-tumour therapy, and the relevance of harnessing the immune system to control cancer has been highlighted in recent years [28,29].

Anti-tumour immune responses can be strongly stimulated by multimodal therapies targeting different aspects of cell killing. In our case, we aimed, on the one hand, to induce immunogenic tumour cell damage while sparing replicating immune system cells (with IMS chemotherapy). On the other hand, we wanted to actively counteract the immune suppression microenvironment within the tumour (PD-1/PD-L1 pathway blockade). We studied relapsing GL261 tumours treated with IMS–TMZ, and found that the upregulated PD-L1 content in tumour tissue is a contributor to TMZ resistance [20]. In this sense, we wondered whether the combination of IMS–TMZ and anti PD-1 antibody in GL261 GB bearing mice would produce more beneficial results in comparison with individual drug use. The results obtained in this study confirmed this possibility (100% cure rate in the combination group), a synergistic effect is likely to result in the enhanced survival of mice treated with combination therapy. One possible explanation for this synergy could be the anti PD-1 antibody blocking PD-L1 binding to PD-1, enhancing T-cell action and reducing the immunosuppressive effects in the tumour microenvironment. Thus, in this case, tumour cell sub-clones expressing a high content of PD-L1 could not suppress anti-tumour immunity and failed to induce tumour escape. This effect would be absent from TMZ monotherapy experiments. Our results are in line with work from other authors [30] which obtained 100% of cured mice with the combination therapy of the same two agents, although with a different protocol of administration.

**Figure 7:**
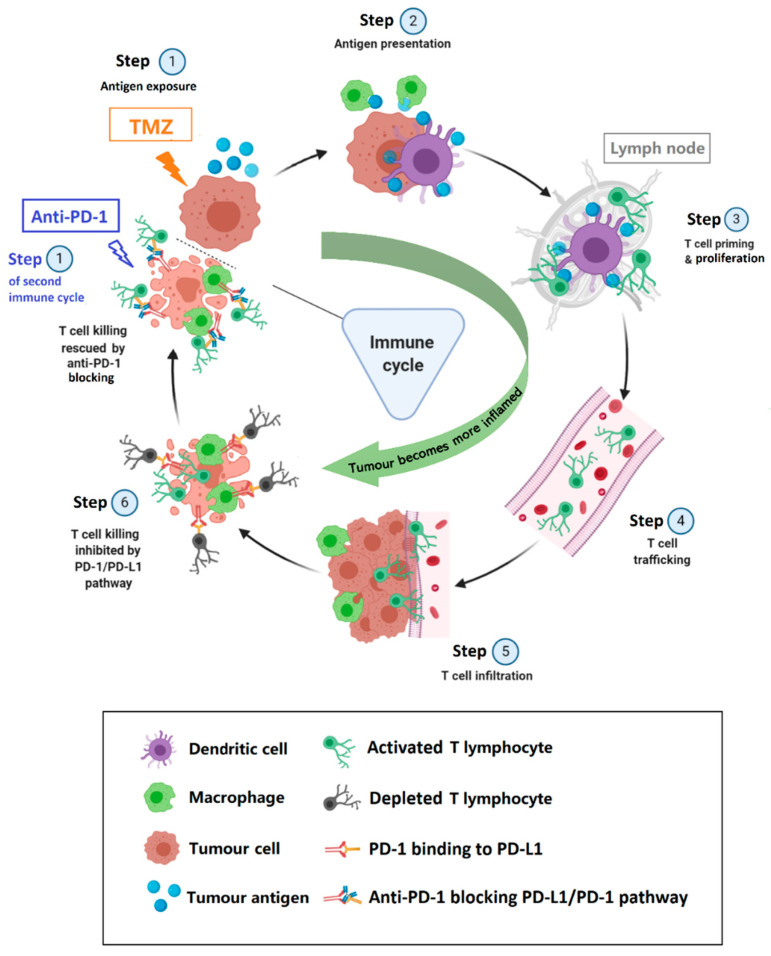
Proposed schema of the cycle for an immune response against a preclinical GL261 GB tumour after two cycles of anti-PD-1/TMZ combined therapy. The cancer-immunity cycle is thought to last around 6 days in the mouse brain and tumour microenvironment: when treated with combination therapy at day 1, tumour cells release and expose immunogenic signals which attract immune cells, e.g., dendritic cells (DCs), macrophages, lymphocytes to the tumour site (Step 1 and 2). Initially, tumour cell killing/damaging mostly relies on the TMZ cytotoxic/cytostatic effect, the immune system is not especially active against the particular tumour cell present. On days 3–5, activated DCs have migrated to the proximal lymph nodes and prime naïve lymphocytes, which start to proliferate (Step3). At day 6 of the cycle, the wave of effector CD8+ T cells peak at the tumour site and efficiently attack the tumour (Step 4–6). However, in this time lapse, tumour cells and macrophages may increase the expression of PD-L1, which binds to PD-1 expressed by T lymphocytes depleting/deactivating them, allowing tumour cells to evade the immune system attack. At this critical juncture, anti-PD-1 administration from the next round of combination therapy (day 1 of the next cycle) may play a key role to rescue and enhance the T lymphocytes’ killing ability.

Another contributing factor to the synergism may come from TMZ contribution. The therapeutic effects of anti-PD-1 in GB have been reported to be associated with immune infiltration [31]. The most commonly used classifications for the patterns of immune cell infiltration include the “inflamed tumour” (rich in tumour-infiltrating lymphocytes, “hot” tumour), the “immune-excluded” (presence of immune cells at the invasive margin but absence of immune cells at the centre of the tumour) and the “immune-desert” phenotype (absence of relevant immune cells at both the periphery and centre of the tumour, “cold” tumour) [32,33]. In our research, IMS–TMZ induced the immunogenic cell death of tumour cells [34] which may trigger host immune system recruitment, and convert the tumour microenvironment from an “immune desert (cold tumour)” to an “inflamed (“hot”) tumour”. This hypothesis is supported by previous results such as increased calreticulin (CRT) exposure after TMZ treatment in GL261 cells [34] and histopathological studies showing an increase in microglia/macrophage in responding tumours after TMZ standard treatment [20]. This being the case, a TMZ treatment effect would recruit tumour infiltrating lymphocytes whose action would be enhanced by the next turn of immune checkpoint inhibitor anti-PD-1 administration (see proposed schema in Figure 7), taking place 6 days after the initial TMZ administration. Accordingly, during the second turn of combined therapy administration, both the TMZ effect recruiting lymphocytes and the CKI treatment would come together producing a maximal effect. In this respect, the volume of all tumours in the combination therapy group decreased drastically after the second therapy administration time, which would agree with the above proposed mechanism.

### 3.2. Finding the Optimal Schedule for Non-Invasive Response Assessment: The Relevance of Tumour Volume at Therapy Starting Time and Dosing Schedule in Anti-PD-1 Monotherapy

When we treated GL261 GB bearing mice with anti-PD-1 monotherapy at the 100 μg dose, the therapeutic effect was not significantly different from that of the isotype control group (23 ± 2.9 days vs. 21.8 ± 3.3 days survival time), which was a disappointing result. Other authors reported that anti-PD-1 at a higher dose and different schedule (500/250 μg) could significantly improve the long-term survival rate (50% mice survived over 100 days) [16]. Therefore, we moved to the higher dose (500/250 μg) of anti-PD-1 monotherapy with our GL261 GB bearing mice. Based on our previous experience, the tumour volume at the therapy starting point could be determinant in the outcome. Accordingly, we explored the impact of initial tumour size on the therapy starting day with the new chosen dosage (500/250 μg from day 11 post-implantation) and found that the cure rate was significantly higher in the small initial tumour group when compared to the large initial tumour group (75 vs. 0% long-term survival). Some studies have pointed out that the tumour mass could probably hamper on its own immune system capability of the host [35] and in this context, our results are not surprising. Importantly, the main premise of cancer immunotherapy is to use/enhance the host immune system to attack tumour cells. Thus, accurate initial tumour volume measurement on the immunotherapy starting day is relevant to understand results and evaluate efficacy. However, most anti-PD-1 therapy studies in preclinical GB models focused on the starting time of administration rather than the initial tumour volume [16,17,18], and even for the same preclinical model/cell line, there could be variations in the tumour development rate. Lacking this information may lead to a loss of accuracy and the misinterpretation of the immunotherapy results in preclinical studies. Thus, determining the tumour volume before the immunotherapy launch is relevant in both preclinical development and clinical trials and would be strongly advised whenever possible. In our hands, a suitable volume to start anti-PD-1 therapy with GL261 tumours is ca. 2.1 ± 1.1 mm^3^, which finally led to the curing of 75% of the animals, allowing ample time for the evaluation of biomarker performance (Section 2.3).

A second relevant question was related to timing in immunotherapy administration. We wondered whether the “every 3 days (E3D)” administration schedule described in [16] was more, less or equally efficient in comparison with “every 6 days (IMS)” for anti-PD-1 (500/250 μg). For that, the therapy starting day described in [16] was maintained for proper comparison. No significant difference in therapy efficacy was found between both groups, indicating that at least for this preclinical model, the same therapeutic effect would be obtained with less frequent administration. The mechanisms underlying here should be related to those described in previous work [20], the whole immune cycle usually takes about six days in the mouse brain [36], thus the administration of anti-PD-1 therapy in synchrony with this cycle would be more fruitful, while an application out of this window would not lead to increased efficacy, although no negative effect would be expected either. Moreover, while providing equivalent anti-tumour efficacy, IMS protocol allows us to reduce the cumulative amount of administered anti-PD-1, reducing the risk of development of dose-dependent autoimmune response [37]. In addition, mouse survival rates (66.6 and 60%) were slightly better than Reardon et al. [16] (50% long-term survival), and also better than another study, with anti-PD-1 given every 7 days [18] (10% long-term survival).

### 3.3. The Oscillatory MRSI Metabolomic Pattern Changes Are Also Confirmed in GL261 GB Treated with Immunotherapy

The classifier of MRSI-based pattern oscillatory changes (TRI oscillation) acting as a potentially useful biomarker for therapy response was initially developed with TMZ-treated cases [25]. One of the fundamental questions raised was whether it would be useful/applicable to assess the efficiency of other therapeutic agents, which would enhance its translational potential. In fact, it has also been shown to be robust to detect tumour response in cyclophosphamide (CPA)-treated mice [38], indicating that the changes observed are not specific to TMZ, but rather linked to local tumour tissue changes during transiently successful treatment. However, CPA is an alkylating agent, similar to TMZ, and the robustness of the source-based classifier has not been checked using therapeutic agents different from chemotherapy. This was assessed for the first time in this study. If our hypothesis was correct, i.e., that TRI oscillations would be a reflection of local immune system action, it should also be observed with anti-PD-1 monotherapy or in combination treatments. Results confirmed the presence of TRI oscillations in GL261 tumours responding to treatment with combination therapy and immunotherapy alone, although the smaller cohort and the variability of the mice population prevent us from generalizing. It is worth noting that responding/control spectra showed the expected metabolic changes already described in [25,26], namely, the regions of lactate/ mobile lipid (ML) (1.33, 4.1 ppm) or polyunsaturated fatty acid (PUFA) (2.8 ppm) showed remarkable differences (see Figure S8 in Supplementary Materials), suggesting that these molecular features are common to different therapeutic strategies provided the immune system is involved in tumour fighting. Moreover, their oscillations matching cancer immune cycle length indicate that proper immune system elicitation is taking place within evaluated tumours.

TRI oscillations (6.6 ± 2.2 days, *n* = 7) were observed in both groups, except by mouse C1479 which did not respond to therapy. After therapy administration, TRI increased from near-zero to high values (79.7 ± 21.4%, *n* = 7) and decayed two days after the TRI peak. In this respect, it should be noticed that the TRI peak maxima is also followed by a reduction in tumour volume in four out of five cases, an effect also observed in IMS–TMZ treated cases [20]. This reproducible behaviour may underlie that a rise in TRI combined with further tumour volume decrease is indicating an active anti-tumour response mediated by the host immune system. Unfortunately, in some cases such tumour volume decrease prevented the assessment of further TRI cycles due to resolution limitations with MRSI approaches.

Indeed, one of the handicaps of the MRSI-based nosological image calculation technique is that small sized tumours do not produce confident segmentation (e.g., case C1446 after day 23 p.i. and C1480 after day 26 p.i.). We named this period the “below threshold detection period” (BTDP) since the semi-supervised source analysis software was not able to properly segment the tumour. However, since small tumours have a trend for better survival or either cure, the metabolomics signature would not be as relevant as in tumours with larger volumes, in which this information is relevant to assess the efficacy of a therapeutic strategy.

Results from previous work suggest that this oscillatory TRI behaviour could serve as a potential immune system efficiency biomarker during the therapy response of GB. Our studies with immunotherapy have highlighted the importance of this biomarker, since the therapeutic effects of anti-PD-1 have been reported to be associated with changes in immune infiltration [31]. It has been seen in case C1479 (Figure S7) that non-oscillating trend or only small, incipient increases in TRI are correlated with the worst outcomes.

It is worth mentioning that in the final stages of tumour evolution in PD cases, high TRI values can be also seen, although without an oscillatory pattern (e.g., case C1479). The single fact that TRI value increases in these cases may indicate that the ‘responding source’ is not only related to a pattern of therapy response but also to local changes due to a rapid tumour expansion. For example, the presence of necrotic, non or low oxygenated (hypoxic) areas that may cause cell death or the activation of the mouse immune system [39,40]. Thus, the “responding” is the most similar source according to the classifier but we cannot discard that it belongs to a fourth, different source until a new mathematical study is eventually performed.

Finally, with anti-PD-1 monotherapy, the observed pattern changes should be essentially due to the action of the host immune system alone, without the confounding effect of chemotherapy. The oscillatory mode detected reinforces the translational potential of the MRSI-based biomarker for patient-tailored GB therapy, including immunotherapy.

### 3.4. IMS-Anti-PD-1/TMZ and Anti-PD-1 Monotherapy Established Varying Degrees of Long-Term Specific Anti-Tumour Immunity

In the present clinical landscape, patients with GB always relapse even after the best accepted therapeutic protocols are applied, including surgical resection. Accordingly, both response improvement and the generation of tumour-specific immunological memory would be crucial to improve prognosis. Thus, we tested how the different treatment modalities had an impact on tumour recurrence after curing mice. A strong immune-memory effect (ca. 100% tumour rejection rate) is observed after GL261 tumour ablation with anti-PD-1 monotherapy, whereas a weaker anti-tumour immune memory capability (50% tumour rejection rate) was found in mice cured by anti-PD-1/TMZ combined therapy.

One exciting feature of immunotherapy is its ability to conduct a dual-phase therapeutic benefit, which initially involves the effective treatment of existing tumours, followed by a successful activation of tumour-specific immune response to fight a possible tumour recurrence. In a clinical study, Ribas and colleagues [41] analysed 102 tumour biopsies obtained from 53 patients treated with an PD-1 antibody (pembrolizumab) by multicolour flow cytometry. They found that the PD-1 blockade therapy enhances the proliferation of T cells, B cells, and myeloid-derived suppressive cells (MDSCs) in the tumour site, being CD8+ memory T cells the main T-cell phenotype in patients with therapy response. In this sense, anti-PD-1 monotherapy in our study probably helped to generate a potent memory CD8+ T cell, effectively (100% tumour rejection rate) protecting cured mice from tumour re-challenge. Moreover, some preclinical studies also reported that anti-PD-1 monotherapy could induce anti-tumour immunological memory in C57BL/6 immunocompetent mice bearing the GL261 tumour [16,30].

Interestingly, weaker anti-tumour immune memory capability (i.e., 50% tumour rejection rate) was found in mice cured by anti-PD-1/TMZ combined therapy. Three of six re-implanted tumours in these cured mice grew after 6 days, although two of them disappeared immediately after repeated IMS-type therapy application. The kinetics of tumour growth after the re-challenge experiment was slower in these tumour-recurrence mice compared to the control mice (mice whose GB tumour grew after re-challenge were shown to be easier to be cured after treatment was re-instated), suggesting that combination therapy had been able to generate at least partial immune memory. Combination therapies are reported in the literature with different outcomes. Authors in [30] reported that favourable immunological effects of anti-PD-1 therapy were abrogated when TMZ was administered 5 days in a row before anti-PD-1 therapy administration. In their studies, the anti-tumour immunological memory was only observed with mice treated with anti-PD-1 monotherapy, which agrees with results described in this study. However, in our work, even the combination therapy was able to mount some immune memory although at a lower rate (50%). These results support the concept of IMS–TMZ as an immune respectful administration protocol: in our case, TMZ did not abrogate the immunological memory provided by anti-PD-1. Furthermore, in another study [19], the authors found that the anti-tumour immunity caused by anti-PD-1 therapy was abrogated by chemotherapy when the latter was administered in a systemic way (intraperitoneal injection in their case), with decreased immune memory in long-term survivors. However, when chemotherapy was administered locally (intra-tumoural), it allowed for persistent immunologic memory generated by anti-PD-1 therapy. This provides us new enlightenment for the combination therapy that is worth considering. In future research, we should not only focus on the therapy administration schedule but also consider different ways of chemotherapy delivery. We should expect that the right therapy combination and the preservation of host immune system functions will result in the improvement of GB outcome, probably even curing some cases, provided they are diagnosed at a relatively early stage, or, perhaps, therapy is given in a neoadjuvant fashion, prior to surgery [13].

It may be relevant to return here to the apparent discrepancy between promising preclinical results after single or combination anti-PD-1 based GB treatment (this work), which would agree with the earlier neoadjuvant use of anti PD-1 [13,14] producing increased patient survival, and the interim report of the CheckMate-548 Phase 3 clinical trial [15]. In this ongoing trial, the addition of *Opdivo* (nivolumab) to the current standard of care b (TMZ and radiation therapy) vs. the standard of care alone did not meet one of its primary endpoints, PFS, in patients with newly diagnosed GB that is O6-methylguanine-DNA methyltransferase (MGMT)-methylated. It is tantalizing to speculate that the radiochemotherapy standard of care treatment (daily TMZ and radiotherapy administration) does not respect the needed time gap to allow for unencumbered lymphocyte amplification, which our IMS protocol does. Accordingly, further clinical trials targeting maximal beneficial effect for patients may want to consider incorporating “immune respectful” time gaps when combining chemotherapy and CKI agents.

### 3.5. Limitations of Our Study

It is wise to recognize that our preclinical study is limited to only one immunocompetent GB model. In this respect, we cannot ignore that GL261 is a moderately immunogenic cell line [42]. Thus, a basal part of the response to therapies may be helped by this basal immunogenicity, like in “hot” tumours [43]. Nonetheless, it seems clear that this basal immunogenicity alone is not able to make C57BL/6 mice resistant to GL261 GB growth, in the absence of IMS–TMZ therapy. It may be relevant to evaluate in future work whether less immunogenic models respond to IMS combined TMZ/anti PD-1, like the CT-2A model [44]. Moreover, other authors favour genetically engineered mice (GEM) models of GB, harbouring defined driver mutations leading to tumour progression [45,46,47], which are claimed to better reproduce human GB development. We hope the robustness of our treatment strategy and the MRSI-based biomarker used for the detection of its efficiency can be put to test also in GEM models of GB by our team or by other groups in the field.

Overall, our results support that (a) the participation of the immune system in response to therapy in preclinical GL261 GB is clear either in monotherapy or combined therapy and (b) our non-invasive MRSI-based therapy response biomarker is clearly related to immune system local action rather than linked to single therapeutic agent local effects.

## 4. Materials and Methods

### 4.1. GL261 Cells

The mouse glioma cell line GL261 was obtained from the Tumour Bank Repository at the National Cancer Institute (Frederick/MD, USA) and was grown as previously described [48].

### 4.2. Animal Model

All animal studies were conducted according to the protocol approved by the local ethics committee (*Comissió d’Ètica en l’Experimentació Animal i Humana*, https://www.uab.cat/etica-recerca/) according to regional and state legislations (protocol CEEAH-3665 approved on 5th February 2018).

A total of 38 C57BL/6 female wild type (wt) mice were used in this study, among them, 35 C57BL/6 GL261 tumour bearing mice were generated for this work. They were obtained from Charles River Laboratories (l’Abresle, France) and housed in the animal facility of the *Universitat Autònoma de Barcelona*. Tumours were induced in C57BL/6 mice by the intracranial stereotactic injection of 100,000 GL261 cells as previously described by us [49]. Tumour size and body weights of mice were measured twice a week and the tumour volumes were followed using T2 weighted (T2w) MRI acquisitions. Mice with the most homogeneous weights and tumour sizes (except cases in Section 2.2) were randomly chosen before therapy was started to make experimental groups.

### 4.3. Animal Treatment

For combination therapy experiments, mice in the anti-PD-1/Temozolomide combined group were given TMZ (Sigma-Aldrich, Madrid, Spain) 60 mg/kg by intragastric administration and anti-PD-1 (Bio X cell, Lebanon, NH, USA) 100 μg/dose via intra-peritoneal injection on the same day, both therapies administered in IMS (every 6 days) from day 11 post-implantation until tumour escape from therapy. Mice in the monotherapy groups received equivalent doses of drugs and control mice received equivalent doses of isotype IgG (Bio X cell, Lebanon, NH, USA) according to the same dosing schedule.

For high dosage (500/250 μg) anti-PD-1 monotherapy experiment, see specific result sections for the detailed anti-PD-1 administration scheme. After treatment, animals meeting endpoint criteria were euthanized by cervical dislocation according to animal welfare protocol advice for ethical reasons. In all the therapy strategies, cured mice were followed up and a re-challenge experiment was carried out (for further details, see Section 4.5).

### 4.4. In Vivo MRI and MRSI Studies

#### 4.4.1. Data Acquisition

MR studies were carried out at the joint NMR facility of the Universitat Autònoma de Barcelona and CIBER-BBN (https://www.ciber-bbn.es, Cerdanyola del Vallès, Spain) Unit 25 of the ICTS NANOBIOSIS (https://www.nanbiosis.es) with a 7T horizontal magnet (BioSpec 70/30, Bruker BioSpin, Ettlingen, Germany) equipped with actively shielded gradients (B-GA12 gradient coil inserted into a B-GA20 gradient system) and a quadrature receive surface coil, actively decoupled from a volume resonator with 72 mm inner diameter. Mice were anesthetized by inhalation of a 0.5–2.0% isoflurane in O2 and placed on the scanner bed prior to the MR study, keeping the respiratory frequency at 60–80 breaths/min. Mice body temperature was controlled using a recirculating water system incorporated to the scanner bed. Animal breathing was monitored constantly with a breathing sensor (SA Instruments, Inc., New York, NY, USA).

#### 4.4.2. MRI Studies

GL261 tumour bearing mice were screened using the Rapid Acquisition with Relaxation Enhancement (RARE) sequence to acquire high-resolution axial T2w images (TR/TEeff = 4200/36 ms) to detect brain tumour presence and track its evolution process. The acquisition parameters for MRI studies can be found in the Supplementary Materials.

##### Tumour Volume Calculation

The manual segmentation of abnormal brain mass in T2w images was performed and tumour volumes were calculated from T2w high resolution horizontal images using the Equation (1):

TV (mm^3^) = [(AS1 × ST) + [(AS2 + (...) + AS10) × (ST + IT)]] × 0.0752 (1)

***Where:*** TV is the tumour volume, surface area (AS) is the number of pixels contained in the region of interest in each slice of the MRI sequence, ST is the slice thickness, IT the inter-slice thickness and 0.0752 the individual pixel surface area in mm^2^. The tumour area was calculated from pixels in each slice with ParaVision 5.1 software. The inter-slice volume was estimated adding the inter-slice thickness to the corresponding slice thickness.

#### 4.4.3. MRSI Studies

The MRSI acquisitions were performed every two days with longitudinally studied mice treated with IMS-anti-PD-1/TMZ combined therapy or IMS-anti-PD-1 monotherapy, starting at day 15–16 post-implantation. MRSI grids were spatially located such that the volume of interest (VOI) included most of the tumour mass as well as normal/peritumoural brain parenchyma (technical details of MRSI acquisitions can be found in the Supplementary Materials and [26]). MRSI acquisitions were forced to be stopped whenever the tumour shrunk down to a small scar, which was below the limit of detection.

#### 4.4.4. MRSI Post-Processing and Pattern Recognition Strategies

MRSI data were post-processed essentially as described in [48]. Briefly, data were initially pre-processed at the MR workstation with ParaVision 5.1, and then post-processed with 3D Interactive Chemical Shift Imaging (3DiCSI) software package version 1.9.17 (Courtesy of Truman Brown, Ph.D., Columbia University, New York, NY, USA) for line broadening adjustment (Lorentzian filter, 4 Hz), zero-order phase correction and exporting the data in American Standard Code for Information Interchange (ASCII) format. Dynamic MRSI processing Module (DMPM), running over MatLab 2013a (The MathWorks Inc., Natick, MA, USA) was used to align all spectra within each MRSI matrix, using the choline signal as reference, 3.21 ppm). The 0–4.5 ppm region of each spectrum in the MRSI matrix was normalized to unit length and exported in ASCII format for performing the PR analysis. No baseline correction was performed in these spectra.

After that, the non-negative matrix factorization (NMF) semi-supervised methodology [25,50,51] was applied for analysing prevailing source signals in the MRSI investigated tumours (normal brain, actively proliferating and responding tumour patterns). From the biochemical viewpoint, the source extraction technique to classify MRS data assumes that in each voxel there is a mixture of heterogeneous tissues and their metabolites from which the contribution of each source can be obtained. Our previously described semi-supervised approach [25], which relies on Convex-NMF for the final source extraction, was used for classifying pixels into normal brain parenchyma, actively proliferating tumour and tumour responding to treatment; and for calculating nosologic maps representing the spatial response to treatment. The green colour is used when the GB responding to treatment source contributes the most, blue for normal brain parenchyma, red for actively proliferating GB and black for undetermined tissue. A more detailed explanation on NMF can be found in the Supplementary Materials and [50,52,53].

#### 4.4.5. Tumour Responding Index (TRI) Calculations

In order to measure the extent of response to treatment using the obtained nosological images, a numerical parameter named TRI was calculated (Equation (2)) [26]:

(2) $$\mathrm{TRI}=\frac{\mathrm{Tumour}\;\mathrm{responding}\;\mathrm{pixels}}{\mathrm{Total}\;\mathrm{tumour}\;\mathrm{pixels}}\times 100$$

TRI is stated as the percentage of green, responding tumour pixels of all grids over the total tumour pixels of all recorded grids.

An adapted set of RECIST criteria (described in Supplementary Materials) was used to classify cases into progressive disease (PD), partial response (PRe) or stable disease (SDi). See the Supplementary Materials for the adapted RECIST criteria.

### 4.5. Rechallenge Experiments

When the tumour was reduced to a stable scar (usually below 2 mm^3^) and no tumour relapse over a 30 day observation period, mice were transiently declared "cured" as in [54]. Cured mice from all groups (anti-PD-1/TMZ combined therapy, *n* = 6; anti-PD-1 monotherapy, *n* = 8) were re-challenged with 100,000 GL261 cells injected into the contralateral hemisphere, symmetric to the initial injection site. A group of three C57BL/6 female wt mice were also implanted as controls, to check for consistency and growth rate in the contralateral side. All mice were monitored (weight + welfare parameters) twice a week and volumetric T2w MRI was acquired twice a week.

### 4.6. Statistical Analysis

Variance homogeneity was assessed with the Levene’s test. Sample distribution was assessed with the Kolmogorov–Smirnov test. A two-tailed Student’s *t*-test for independent measurements was used for comparisons, for samples of equal or different variances (depending on the Levene’s test result). Comparisons of survival rates were performed with the Log-Rank test. The significance level for all tests was *p* < 0.05.

## 5. Conclusions

Suitable combination of oncology and immunology knowledge will pave the way for future GB treatment and improve patient outcome. We explored in an immune-competent, aggressive GB preclinical model how using the direct mitigation of the tumour-based immune suppression microenvironment (PD-1/PD-L1 pathway blockade) either as monotherapy or in combination with TMZ, produced outstanding results, not achieved before with comparable treatments. We also proved, for the first time, that the metabolomic spectral patterns spotted by our MRSI-based surrogate biomarker of therapy response were also observed using anti-PD-1 monotherapy, in agreement with these changes being, at least partially, contributed by the local immune system action against tumour cells. This finding confirms that the MRSI-derived biomarker is not linked to a given therapeutic agent or protocol and should be investigated in any therapeutic approach for GB involving host immune system activation. The possible translation of these protocols would allow to assess, in an early and confident way, therapy efficacy and help in patient management. Further studies may be needed in order to fully characterize the molecular/cellular changes related to the spectral pattern changes detected. Last, but not least, such an imaging biomarker could be useful in the follow-up of other brain pathologies in which the immune system can play a role such as a stroke or neurodegenerative diseases.

## Figures and Tables

**Table 1:** Cured mice distribution in different groups investigated in this section.

Treatment	IMS-Anti-PD-1/TMZ	IMS-Anti-PD-1 Monotherapy
Mice code	C1386	C1440
	C1398	C1441
	C1402	C1442
	C1431	C1437
	C1433	C1438
	C1446	C1454
		C1480
		C1484
Number of animals	6	8

**Table 2:** Comparison of the GL261 tumour take rates between primary tumour implantation, and GL261 re-challenge in IMS-anti-PD-1/TMZ and anti-PD-1 monotherapy cured mice.

	Primary Tumour Implantation wt Control Mice	Tumour Re-Challenge IMS-Anti-PD-1/TMZ Cured Mice	Tumour Re-Challenge Anti-PD-1 Monotherapy Cured Mice
Mice with growing tumour	3	3	0
Mice with upfront tumour rejection	0	3	8
Tumour rejection rate	0%	50%	100%

## Supplementary Materials

Supplementary Materials can be found at https://www.mdpi.com/1422-0067/21/22/8775/s1.

## Abbreviations

3D	Three dimensional
AS	Surface area
ASCII	American Standard Code for Information Interchange
BTDP	Below threshold detection period
CKI	Check point inhibitors
CNS	Central nervous system
CPA	Cyclophosphamide
CRT	Calreticulin
DCs	Dendritic cells
E3D	Every 3 days dosing schedule
GABRMN	*Grup d’Aplicacions Biomèdiques de la Ressonància Magnètica Nuclear*
GB	Glioblastoma
IgG	Immunoglobulin G
IMS	Immune-Enhancing Metronomic Schedule
IT	Inter-slice thickness
MDSCs	Myeloid-derived suppressive cells
ML	Mobile Lipid
MRI	Magnetic resonance imaging
MRS	Magnetic Resonance Spectroscopy
MRSI	Magnetic resonance spectroscopic imaging
NMF	Non-negative matrix factorization
NMR	Nuclear Magnetic Resonance
OSs	Overall survival
PD	Progressive disease
PD-1	Programmed cell death-1
PD-L1	Programmed cell death ligand-1
PFS	Progression free survival
p.i.	Post-implantation
PRe	Partial response
PUFA	Polyunsaturated fatty acid
RARE	Rapid Acquisition with Relaxation Enhancement
RECIST	Response Evaluation Criteria in Solid Tumours
SDi	Stable disease
ST	Slice thickness
T2w	T2-weighted MRI
TEeff	Effective echo time
TMZ	Temozolomide
TR	Recycling time
TRI	Tumour Responding Index
TV	Tumour Volume
VOI	Volume of interest
wt	Wild type
