# Supplementary material for: Anti-PD-1 Immunotherapy in Preclinical GL261 Glioblastoma: Influence of Therapeutic Parameters and Non-Invasive Response Biomarker Assessment with MRSI-Based Approaches
Source: Int J Mol Sci. 2020 Nov 20;21(22):8775. doi: 10.3390/ijms21228775 (PMC7699815; doi:10.3390/ijms21228775)
Supplement: Supplementary file 1 [file ijms-21-08775-s001.pdf]

Supplementary Material for:

Anti-PD-1 Immunotherapy in Preclinical GL261 Glioblastoma: Influence of Therapeutic Parameters and Non-Invasive Response Biomarker Assessment with MRSI-Based Approaches

Shuang Wu, Pilar Calero-Pérez, Carles Arús, Ana Paula Candiota

SUPPLEMENTARY MATERIALS

MRI studies

The acquisition parameters were as follows: turbo factor: 8, field of view (FOV): 19.2 × 19.2 mm, matrix size (MTX): 256 × 256 (75 × 75 μm/pixel), number of slices: 10, slice thickness (ST): 0.5 mm, inter-slice thickness (IT): 0.6 mm, number of averages (NA): 4, total acquisition time (TAT):6 min and 43 s. The acquired MRI data were processed on a Linux computer using software ParaVision 5.1 (Bruker BioSpin GmbH, Ettlingen, Germany).

MRSI studies

Consecutive 14 ms echo time (TE) MRSI with point-resolved spectroscopy (PRESS) localization grids were acquired individually across the tumour, using as a reference T2w high resolution images, as described in previous work [1]. First upper (dorsal) grid (Grid 1) had a matrix size of 10 × 10. Then, Grid 2 was acquired 1 mm below Grid 1 with a matrix size of 12 × 12. Grid 3 was acquired 1 mm below Grid 2, also with a matrix size of 12 × 12. Finally, if the tumour volume was not completely covered with three grids, a final Grid 4 was acquired 1 mm below Grid 3 with a matrix size of 10 × 10.

Acquisition parameters for all grids were: FOV, 17.6 mm × 17.6 mm; VOI in Grids 1 and 4 was 5.5 mm × 5.5 mm × 1.0 mm. VOI in Grids 2 and 3 was 6.6 mm × 6.6 mm × 1.0 mm. ST, 1 mm; TR, 2500 ms; sweep width (SW), 4006.41 Hz; NA, 512; TAT, 21 min 30 s each grid. Water suppression was performed with variable power and optimised relaxation delay (VAPOR), using a 300 Hz bandwidth. Linear and second order shims were automatically adjusted with a fast automatic shimming technique by mapping along projections (FASTMAP) in a 5.8 mm × 5.8 mm × 5.8 mm volume which contained the VOI region. Six saturation slices (ST, 10 mm; sech-shaped pulses: 1.0 ms/20250 Hz) were positioned around the VOI to minimize outer volume contamination in the signals obtained.

MRSI postprocessing: outline of NMF methods

In general, NMF methods belong to a group of multivariate data analysis techniques designed to estimate meaningful latent components, also known as sources, from non-negative data. Standard NMF methods decompose a given data matrix "X" into two non-negative matrices: the sources ("S") and the mixing matrix ("A"). The differences between NMF methods are given by the different cost functions used for measuring the divergence between X and S*A. A more recent variant of NMF that is also able to handle negative data, namely convex-NMF, was used in this work [2]. Convex-NMF has been proven to be able to identify a reduced number of sources that can be confidently recognized as representing brain tumour/tissue types in a way that other source extraction methods, including other NMF variants, cannot detect with the same degree of specificity [3,4].

Adapted RECIST criteria

Classification of adapted RECIST criteria was applied as follows: progressive disease (PD): 20% increase with respect to the smallest tumour volume so far. Partial response (PRe): tumour decrease by 30%, taking into account the biggest volume so far. Stable disease (SDi): less than 20% increase and no more than 30% decrease in tumour volume.

C1539 anti-PD-1/TMZ combination therapy

Case C1539 was analysed by MRI and multi-slice 3D MRSI from day 15 until day 31 p.i. MRSI acquisitions were forced to be stopped from day 31 p.i. since the C1539 tumour had shrunk down to a 8.45 mm^3 scar, entering the BTDP. Therapy was administered on days 11, 17, 23, and 29 p.i., and was maintained in IMS until the animal was considered cured. The relationship between tumour volume and TRI evolution accompanied by the corresponding nosological images are shown in Figure S4.

In this case, TRI increased from 8.9 % at day 15 p.i. to 93.3 % at day 21 p.i. and decreased to 52.3 % at day 25 p.i., then a further increase to 94.1 % was measured at day 27 p.i. After this period, the tumour entered a BTDP period so in total two clear TRI peaks were observed.

The tumour volume of mouse C1539 grew until day 19 p.i., when abundant responding tumour pixels started to be observed. Then, along with the presence of TRI peaks, the tumour volume dropped by 86.4 % between day 19 p.i. and day 29 p.i., probably due to immune system sustained attack onto the tumour leading to shrinkage. Two clear TRI cycles were observed in this case, each 4 days in length.

C1540 anti-PD-1/TMZ combination therapy

Case C1540 was analysed by MRI and multi-slice 3D MRSI from day 15 until day 35 p.i. MRSI acquisitions were forced to be stopped from day 35 p.i. since C1540 tumour had shrunk down to a 6.13 mm^3 scar, entering the BTDP. Therapy was administered at days 11, 17, 23, and 29 p.i., and was maintained in IMS until the animal was considered cured. The relationship between tumour volume and TRI evolution accompanied by the corresponding nosological images are shown in Figure S5.

In this case, TRI increased from 7.2 % at day 15 p.i. to 64.5 % at day 17 p.i. and decreased slightly to 57.9 % at day 19 p.i., then a further increase to 96.4 % was measured at day 26 p.i. After this period, the tumour entered a BTDP period so in total two clear TRI peaks were observed.

The tumour volume of mouse C1540 grew until day 17 p.i., when abundant responding tumour pixels started to be observed. Then, along with the presence of TRI peaks, the tumour volume dropped by 89.3 % between day 17 p.i. and day 35 p.i., probably due to immune system sustained attack onto tumour leading to shrinkage. Two clear TRI cycles were observed, in this case of 6 days and 8 days in length, respectively.

C1484 anti-PD-1 monotherapy

C1484 was analysed by MRI and multi-slice 3D MRSI from day 16 until day 28 p.i., when MRSI acquisitions were forced to be stopped from day 28 p.i. due to technical issues related to MR coil availability during this time period. Therapy was administered at days 6, 12, 18, 24, 30 and 36 p.i., and was continued to be given in IMS until the tumour escaped from therapy. The relationship between TRI and tumour volume as well as the corresponding nosological images are shown in Figure S6.

In this case, there was no significant shrinkage during tumour evolution, however, according to the adapted RECIST criteria, two SDi stages of tumour growth were observed during the MRSI analysis

period (16–20 days p.i.) along the TRI peaks. Moreover, the significantly longer survival time of C1484 also distinguished it from non-responding cases.

One clear TRI oscillation was observed between days 16 and 20 p.i.; such a TRI peak appeared on day 18 p.i., 6 days after the second therapy administration. The lack of MRSI information from day 26 p.i. and beyond due to technical issues prevented us to check whether the second TRI rise would be followed by a minimum (second peak). However, the uncontrolled tumour volume increase does not seem to point towards this direction.

C1479 anti-PD-1 monotherapy

Case C1479 was analysed by MRI and multi-slice 3D MRSI from day 16 until day 26 p.i. when it was euthanized for humanitarian reasons since the tumour volume reached 163.4 mm^3. Therapy was administered at days 6, 12, 18 and 24, right after MRSI analysis. The relationship between tumour volume and TRI evolution accompanied by the corresponding nosological images is shown in Figure S7.

The tumour evolution in this case was classified as PD throughout the period of MRSI analysis, except for a brief interval between days 24 and 26 p.i., the overall trend being similar to an untreated tumour with only slightly longer survival. The TRI oscillatory pattern is not observed in this case. The small variation observed at day 18 p.i. could be attributed to experimental variation, since a TRI cycle was defined provided a change between maximum and minimum TRI values was above 10%, and in this case, variation was only 2.9%.

SUPPLEMENTARY FIGURES:

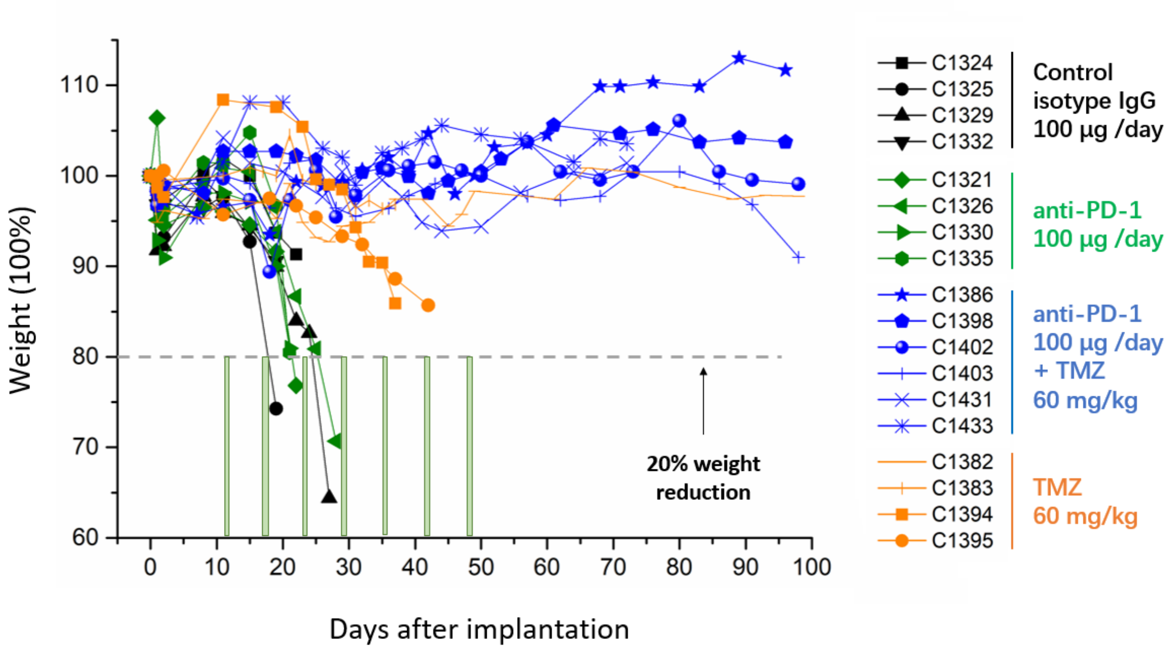

Figure S1. Body weight of the mice treated with control isotype murine IgG 100 µg/day (n = 4, black lines), anti-PD-1 100 µg/day (n = 4, green lines), anti-PD-1 100 µg/day combined with TMZ 60 mg/kg (n= 6, blue lines) and TMZ 60 mg/kg alone (n = 4, orange lines), the transparent green column indicates therapy administration time points.

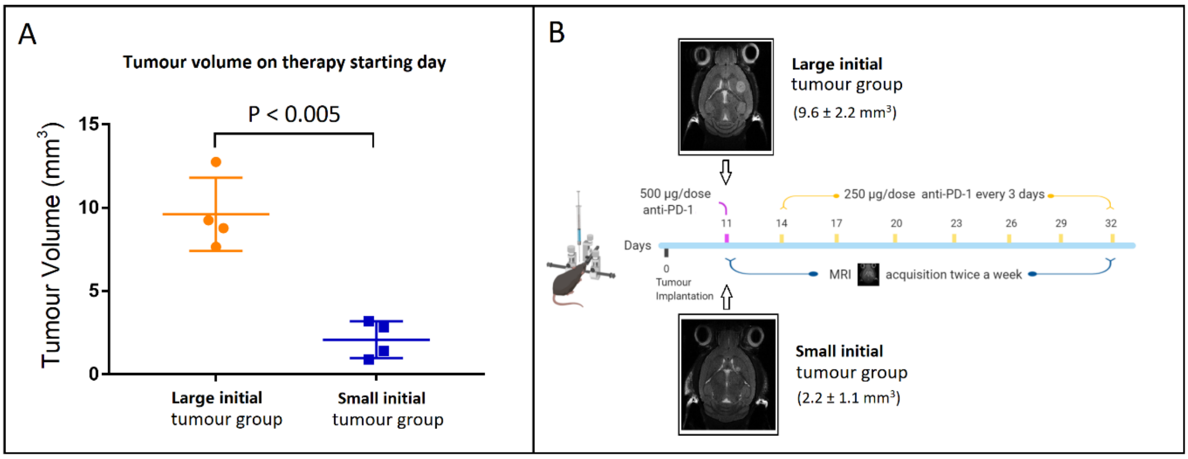

Figure S2. Mice bearing different sized tumours at same post-implantation days and treated with same anti-PD-1 dosing schedule. (**A**) Distribution of tumour volume on the therapy starting day, large initial tumour volume group (orange dots, 9.6 ±2.2 mm³, n = 4), small initial tumour volume group (blue dots, 2.1 ± 1.1 mm³, n = 4). Significant differences (p < 0.005) were found between the two groups with the Student's t-test. (**B**) Experimental treatment schedule and representative T2w tumour images of cases at the therapy starting day.

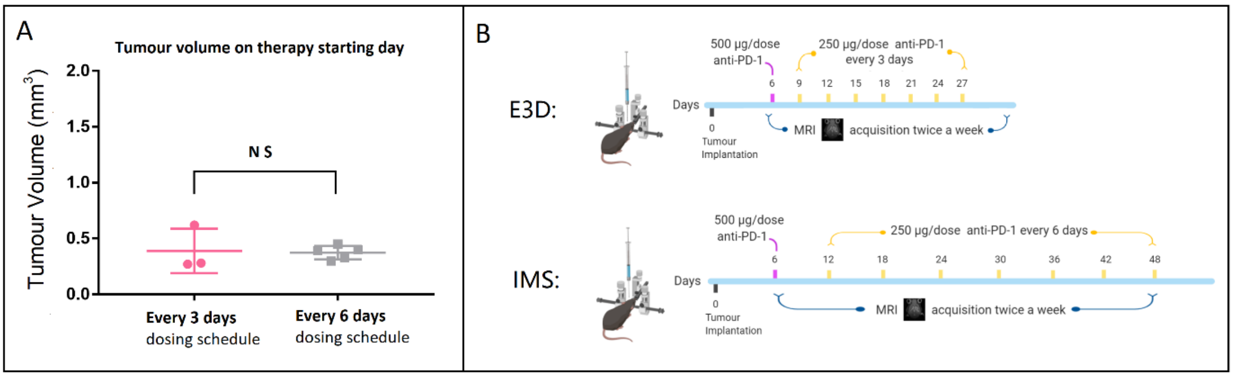

Figure S3. Mice bearing similar sized tumours at same post-implantation days and treated with different anti-PD-1 dosing schedule. (A) Distribution of tumour volume on the therapy starting day, every 3 days dosing schedule group (pink dots, 0.4 ± 0.2 mm^3, $n = 3$), every 6 days group (grey dots, 0.4 ± 0.1 mm^3, $n = 5$). No significant differences ($p > 0.05$) were found between the two groups with Student's t-test. (B) Experimental schedule for the administration of anti-PD-1 (500/250 µg) every 3 day (E3D) and IMS.

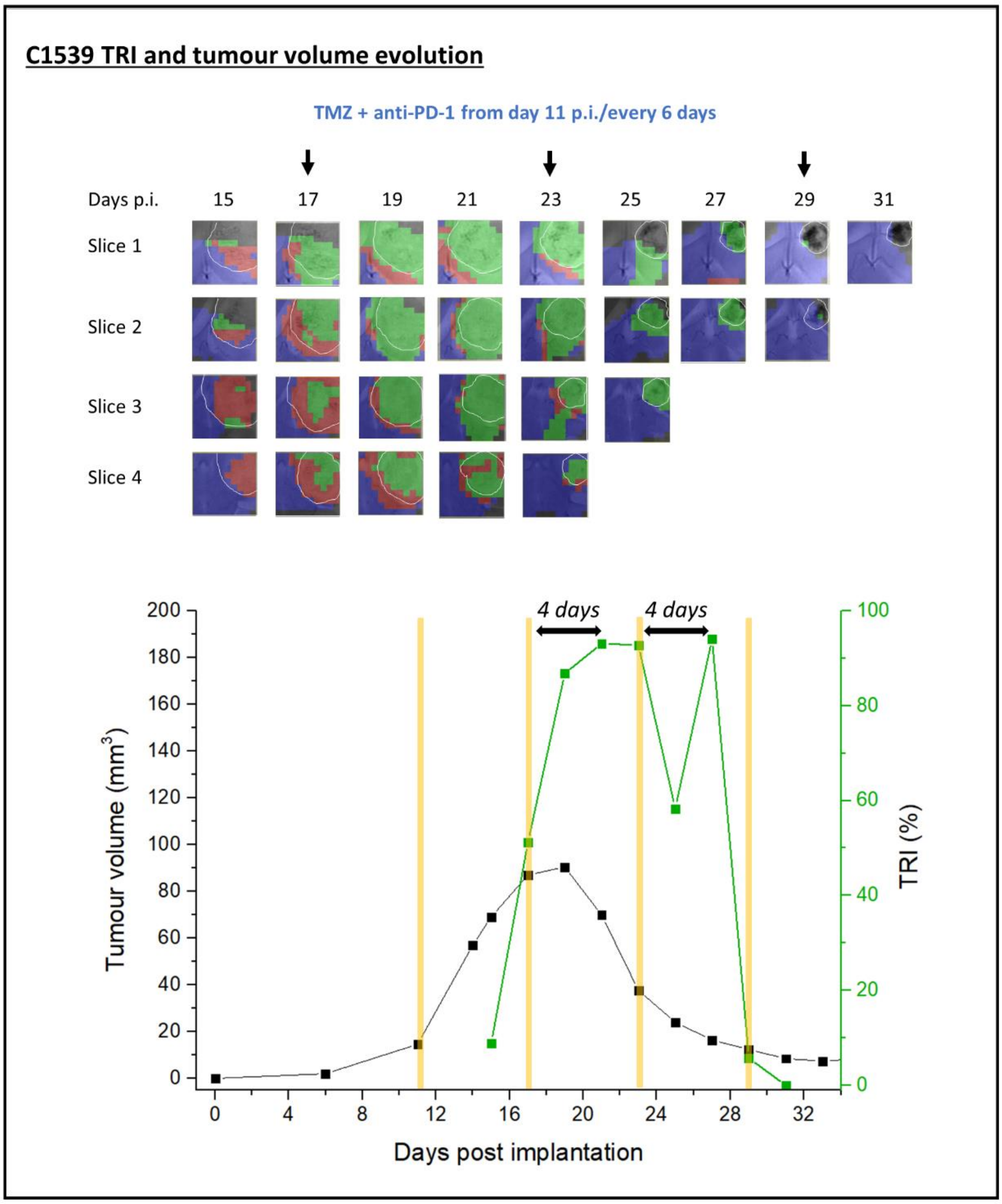

Figure S4. Nosological images and the graphical representation of the tumour volume evolution for the tumour region in case C1539. Tumour volume in mm^3 (black line, left axis) and the percentage of green, responding pixels (TRI) obtained taking into account the total pixels counting (green line, right axis). In the upper part of every image, the chosen time points show the evolution of the nosological images in one to four rows of colour-coded grids superimposed to the T2w-MRI for each slice. Vertical arrows indicate days of therapy administration. In the bottom graph, yellow columns indicate anti-PD-1/TMZ combination therapy administration days. TRI cycle duration (therapy administration to next peak maxima) are highlighted in the image.

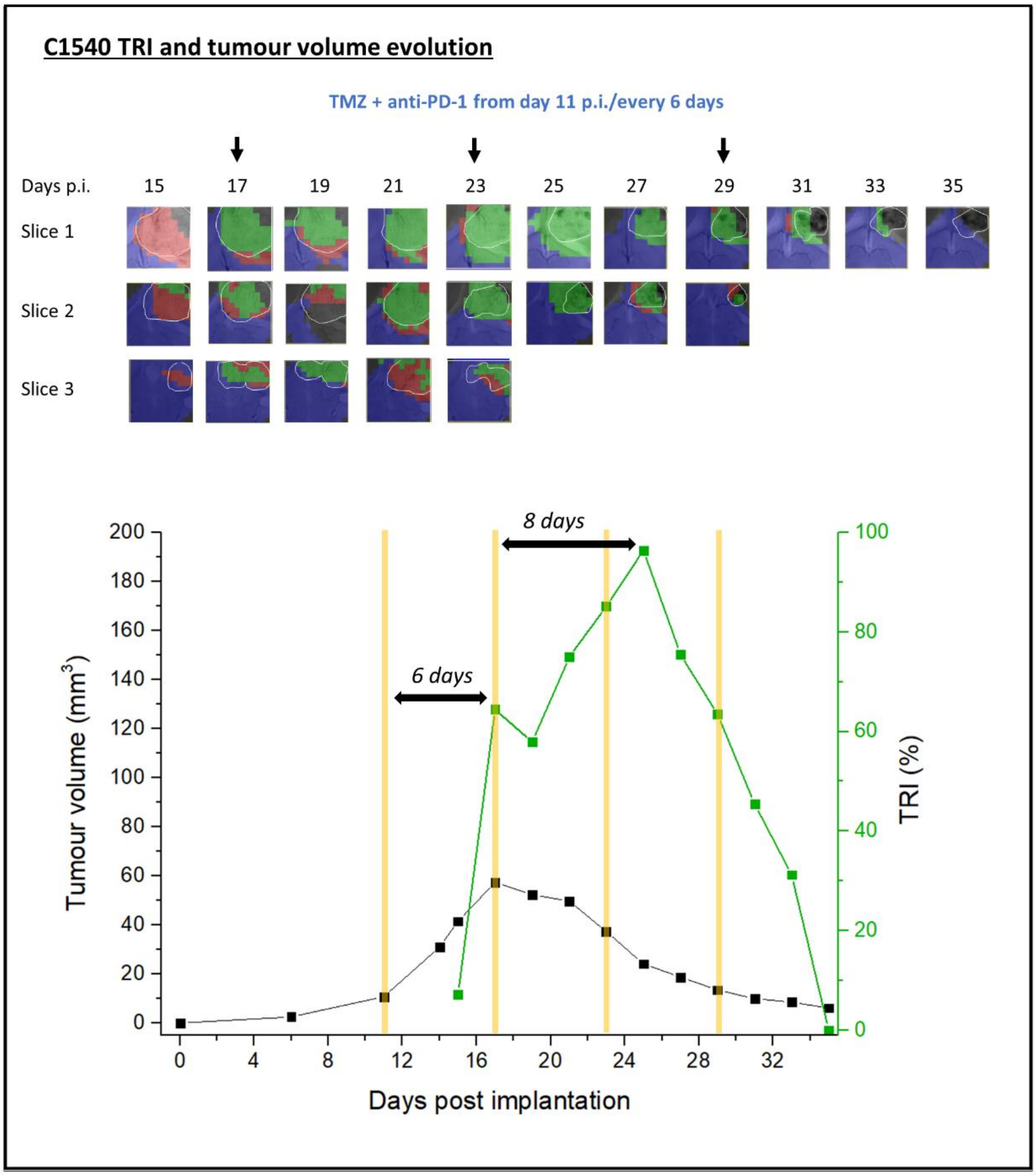

Figure S5. Nosological images and the graphical representation of the tumour volume evolution for the tumour region in case C1540. Tumour volume in mm^3 (black line, left axis) and the percentage of green, responding pixels (TRI) obtained taking into account the total pixels counting (green line, right axis). In the upper part of every image, the chosen time points show the evolution of the nosological images in one to three rows of colour-coded grids superimposed to the T2w-MRI for each slice. Vertical arrows indicate days of therapy administration. In the bottom graph, yellow columns indicate anti-PD-1/TMZ combination therapy administration days. TRI cycle duration (therapy administration to next peak maxima) are highlighted in the image.

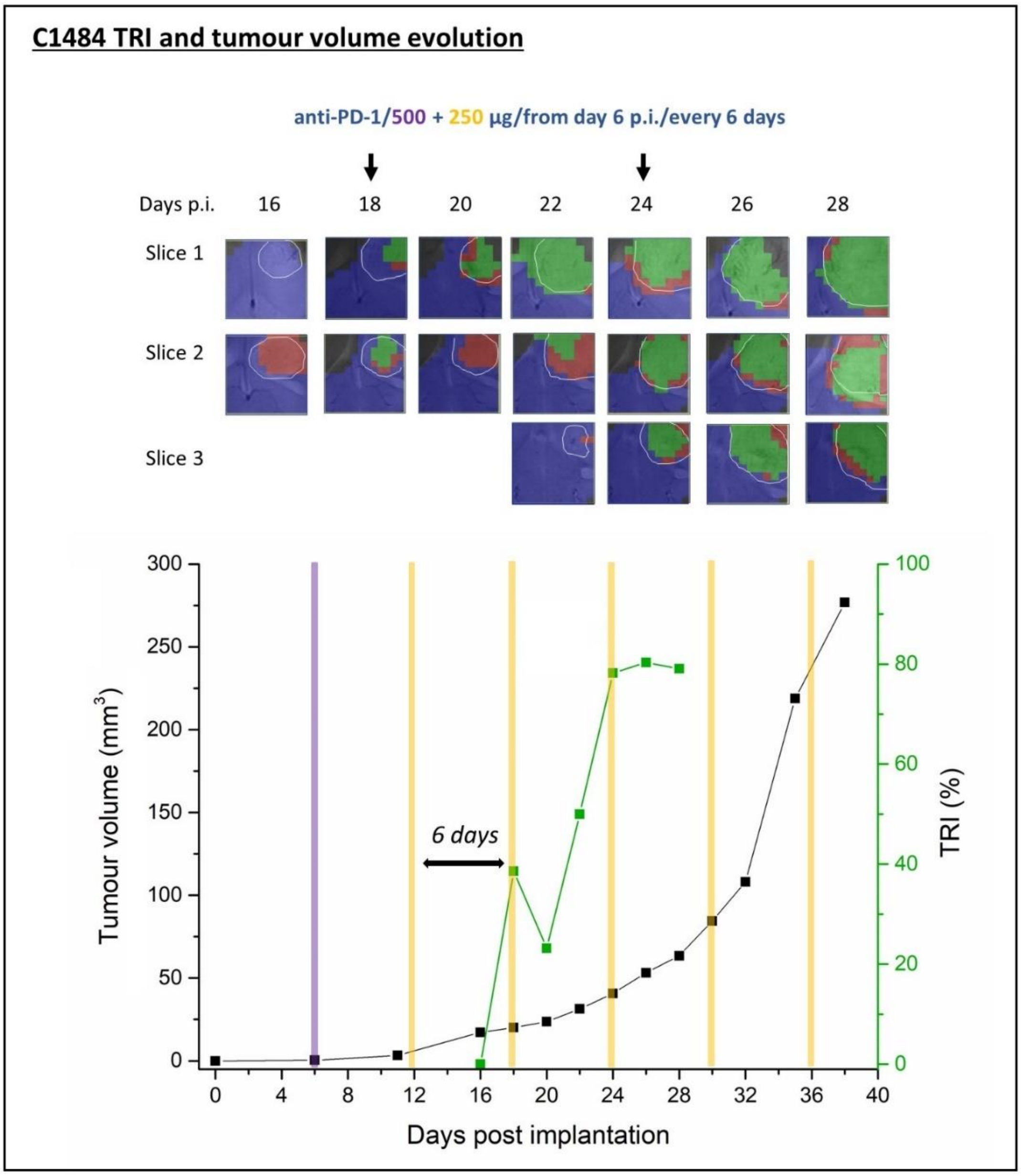

Figure S6. Nosological images and the graphical representation of the tumour volume evolution for the tumour region in the IMS-anti-PD-1 treated case C1484. Tumour volume in mm^3 (black line, left axis) and the percentage of green, responding pixels (TRI) obtained taking into account the total pixels counting (green line, right axis). In the upper part of every image, the chosen time points show the evolution of the nosological images in two to three rows of colour-coded grids superimposed to the T2w-MRI for each slice. Vertical arrows indicate days of therapy administration. In the graph below, the purple and yellow column (500 µg/dose and 250 µg/dose separately) indicate anti-PD-1 administration days. TRI cycle duration (therapy administration to next peak maxima) are highlighted in the image.

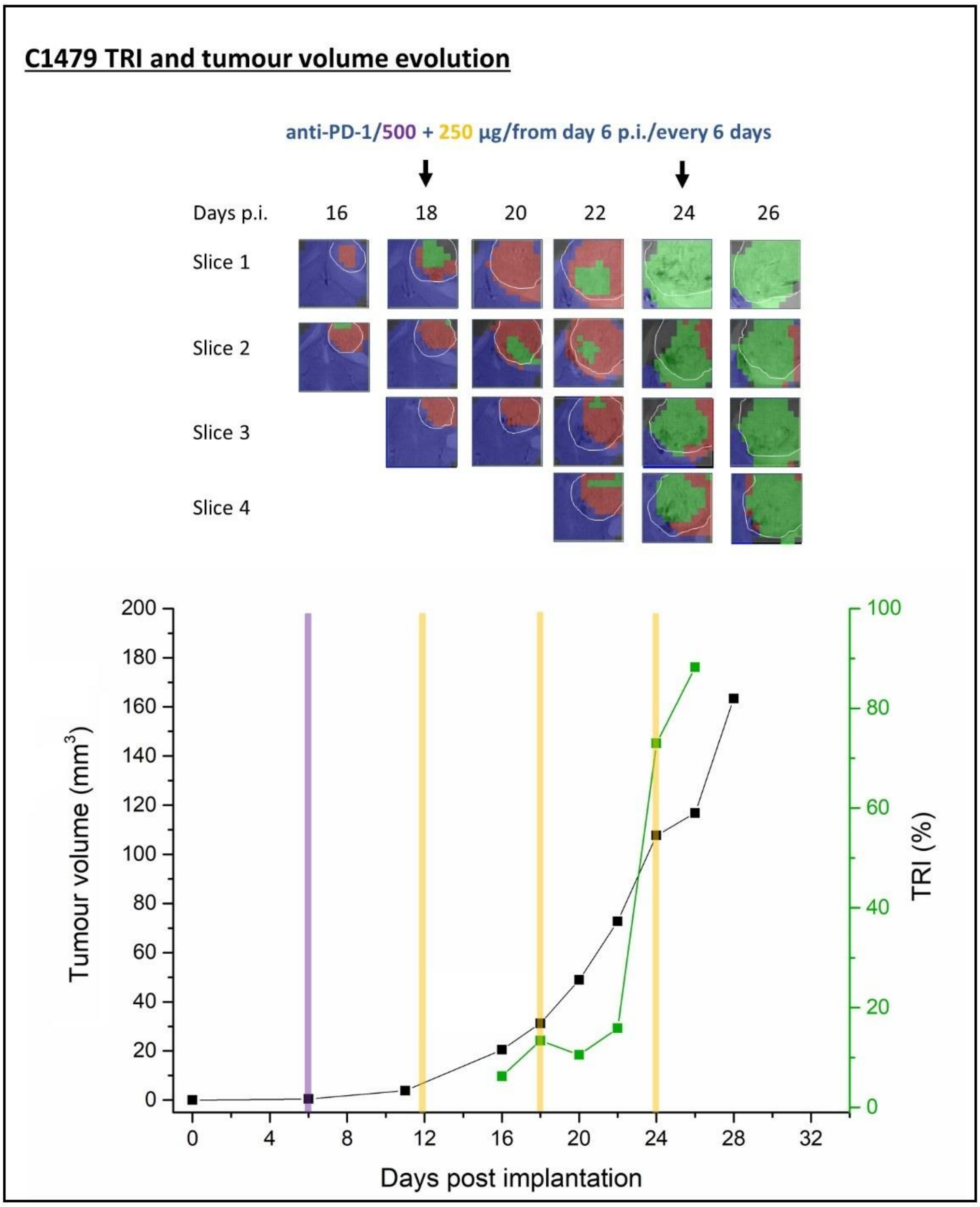

Figure S7. Nosological images and the graphical representation of the tumour volume evolution for the tumour region in the case C1479. Tumour volume in mm^3 (black line, left axis) and the percentage of green, responding pixels (TRI) obtained taking into account the total pixels counting (green line, right axis). In the upper part of every image, the chosen time points show the evolution of the nosological images in two to four rows of colour-coded grids superimposed to the T2w-MRI for each slice. Vertical arrows indicate days of therapy administration. In the bottom graph, the purple and yellow column (500 µg/dose and 250 µg/dose separately) indicate anti-PD-1 administration days. No TRI cycle was observed in this case.

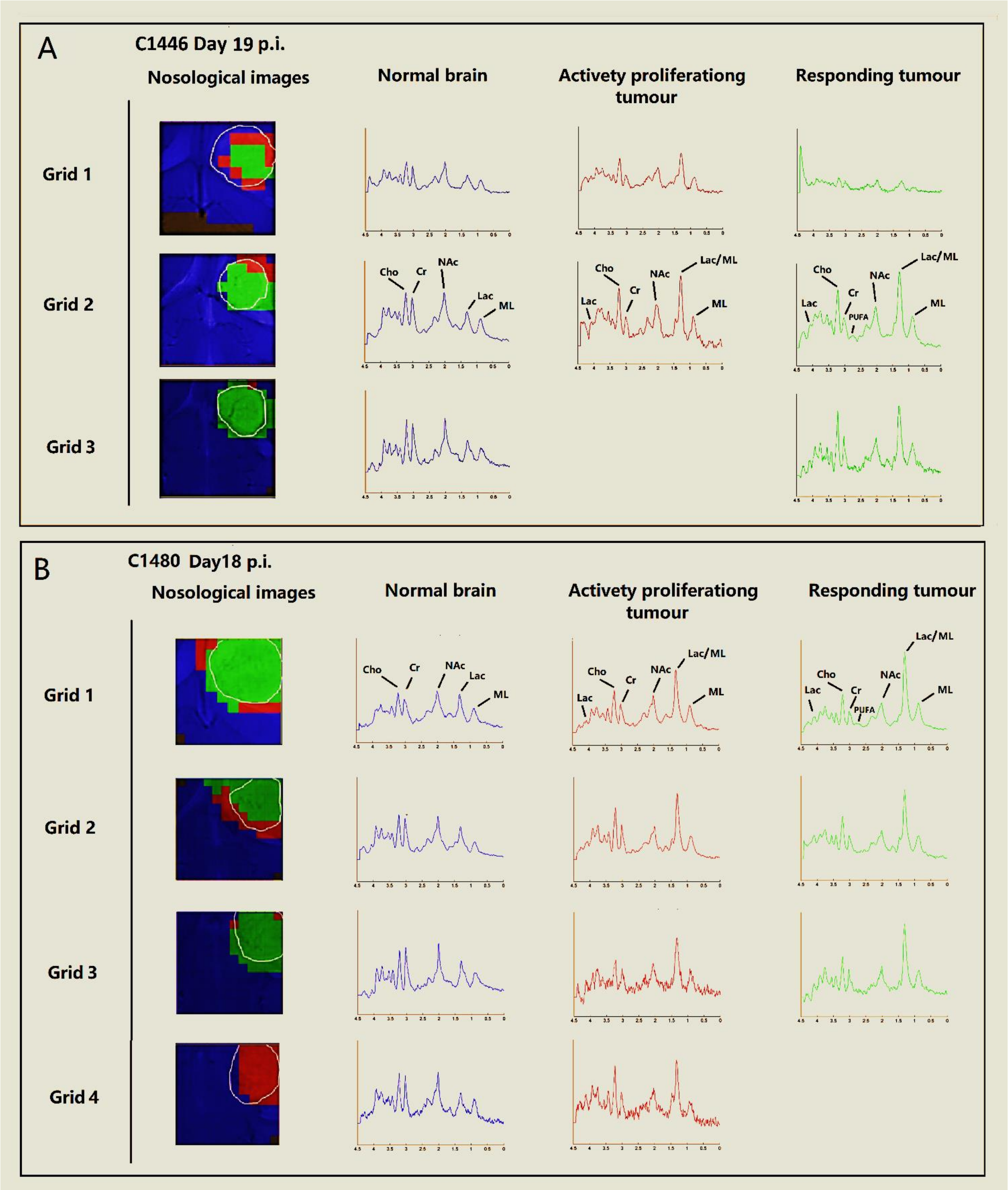

Figure S8. Examples of mean spectra calculated from the chosen zones of the nosological images classified as normal brain parenchyma (blue), actively proliferating tumour (red) and responding tumour (green). (**A**) IMS-anti-PD-1/TMZ case C1446: normal brain (*n* = 298 pixels), actively proliferating tumour (*n* = 18) and responding tumour (*n* = 46). (**B**) IMS-anti-PD-1 treated case C1480: normal brain (*n* = 331 pixels), actively proliferating tumour (*n* = 49) and responding tumour (*n* = 108). Cho= choline, Cr= creatine, NAc= N-acetyl containing compounds, Lac= lactate, ML= mobile lipids. As expected, the tumour zones present a higher Cho/Cr and Cho/NAc ratio in comparison with normal brain parenchyma and higher Lac/ML signals. Still, responding zones present a more noticeable 2.8 ppm signal, compatible with a polyunsaturated fatty acids (PUFA) chemical shift, although differences among tissue types/response status are distributed all along the spectral pattern.

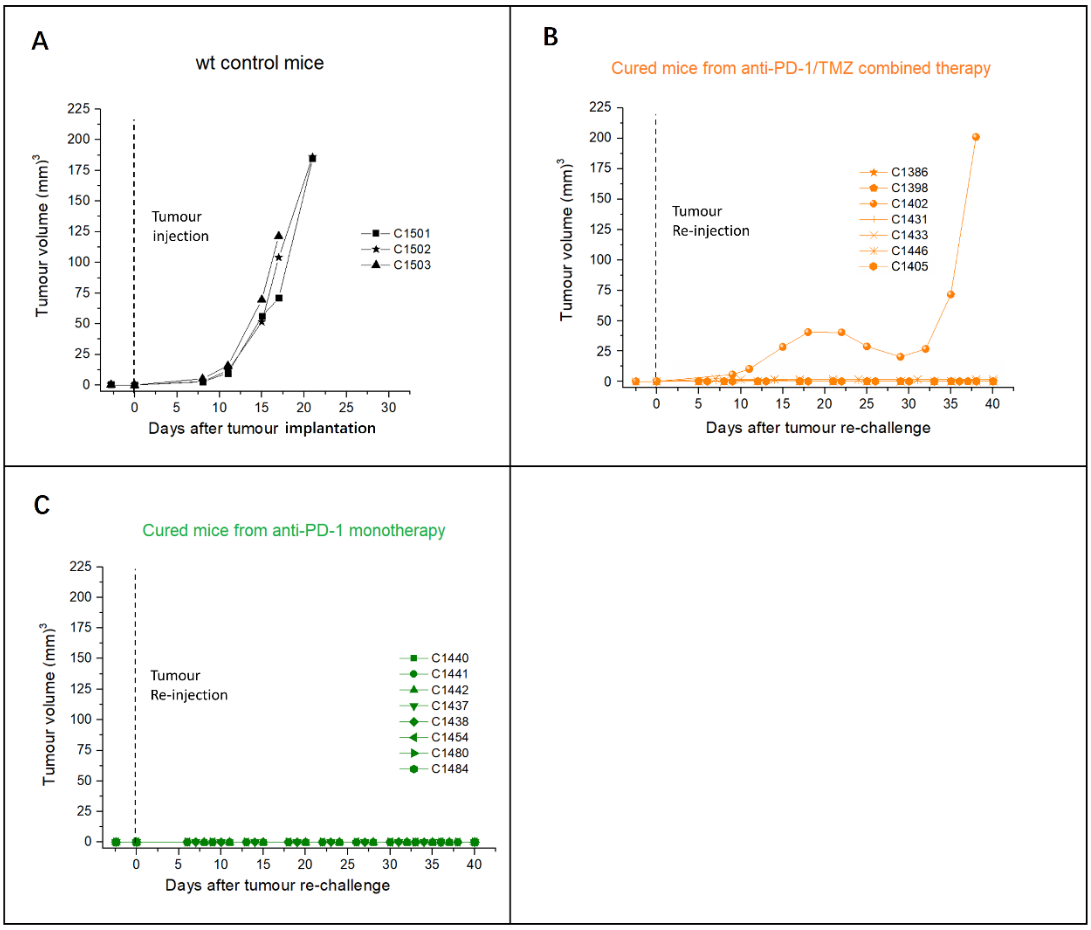

Figure S9. Tumour volume evolution after the re-challenge experiment with GL261 cells in cured mice. (**A**) All wt control mice developed rapidly growing GL261 tumours, as expected. (**B**) In IMS-anti-PD-1/TMZ combined therapy group, one cured mouse (C1402) exhibited an initial transient response followed by tumour regrowth and eventually died due to the growth of the re-implanted tumour, meanwhile two cured mice (C1431 and C1433) had a very small volume (2.15 mm^3 maximal volume) transient tumour growth 7 days after implantation, which was eliminated after 2 doses of IMS-anti-PD-1/TMZ therapy. (**C**) Mice cured from anti-PD-1 monotherapy rejected tumour right away, showing no tumour growth at any time point.

SUPPLEMENTARY TABLES:

Table S1. Evolution of treated GL261 cases considering TRI and tumour volume changes over time. Classification of the adapted RECIST criteria were applied as follows: progressive disease: 20% increase with respect to the smallest tumour volume so far. Partial response: tumour decrease of 30% taking into account the biggest volume so far. Stable disease: less than 20% of increase and no more of 30% decrease.

Group	Case	Day p.i.	TRI behaviour	Classification of RECIST criteria
IMS-anti-PD-1/TMZ	C1446	15–17	TRI cycle	Stable disease
		17–23		Partial Response
		23–29	BTDP	Stable disease
	C1539	17–19	TRI cycles	Stable disease
		19–25		Partial Response
		25–29		Partial Response
		29–31	BTDP	Stable disease
	C1540	17–19	TRI cycles	Stable disease
		19–33		Partial Response
		33–35	BTDP	Stable disease
IMS-anti-PD-1	C1479	16–26	No cycle	Progressive disease
	C1480	16–20	TRI cycle	Stable disease
		20–26		Partial Response
		26–34	BTDP	Stable disease
	C1484	16–20	TRI cycles	Stable disease
		20–28	No cycle	Progressive disease

SUPPLEMENTARY REFERENCES:

1. Arias-Ramos, N.; Ferrer-Font, L.; Lope-Piedrafita, S.; Mocioiu, V.; Julià-Sapé, M.; Pumarola, M.; Arús, C.; Candiota, A.P. Metabolomics of therapy response in preclinical glioblastoma: A multi-slice MRSI-based volumetric analysis for noninvasive assessment of temozolomide treatment. *Metabolites* **2017**, *7*, 1–29, doi:10.3390/metabo7020020.
2. Ding, C.H.Q.; Tao Li; Jordan, M.I. Convex and Semi-Nonnegative Matrix Factorizations. *IEEE Trans. Pattern Anal. Mach. Intell.* **2010**, *32*, 45–55, doi:10.1109/TPAMI.2008.277.
3. Ortega-Martorell, S.; Lisboa, P.J.G.; Vellido, A.; Simões, R. V.; Pumarola, M.; Julià-Sapé, M.; Arús, C. Convex Non-Negative Matrix Factorization for Brain Tumor Delimitation from MRSI Data. *PLoS One* **2012**, *7*, e47824, doi:10.1371/journal.pone.0047824.
4. Ortega-Martorell, S.; Lisboa, P.J.G.; Vellido, A.; Julia-Sape, M.; Arus, C. Non-negative Matrix Factorisation methods for the spectral decomposition of MRS data from human brain tumours. *BMC Bioinformatics* **2012**, *13*, 38, doi:10.1186/1471-2105-13-38.
